# Community and Population Characteristics and Future Potential Habitats Under Climate Change of *Juniperus* Species in Yunnan, Southwestern China

**DOI:** 10.3390/plants14172754

**Published:** 2025-09-03

**Authors:** You-Cai Shi, Qing Chen, Min-Rui Du, Shu-Li Xiao, Shuai-Feng Li, Xiao-Fan Wang, Qiao Li, Cindy Q. Tang

**Affiliations:** 1 College of Ecology and Environmental Science, Yunnan University, Building #3, Guozhong Dalou, Chenggong Campus, Dongwaihuan South Road, University Town, Chenggong New District, Kunming 650504, China; shijian.2012@outlook.com (Y.-C.S.); 18314595772@163.com (Q.C.); marduk.minrui@outlook.com (M.-R.D.); xiaoshuli1995@outlook.com (S.-L.X.); xfwangynu@163.com (X.-F.W.); 18487264306@163.com (Q.L.); 2 Institute of Highland Forest Science, Chinese Academy of Forestry, Kunming 650224, China; shuaifengli@163.com

**Keywords:** *Juniperus*, community structure, population status, species diversity, phylogenetic relatedness, regeneration, climate change, conservation

## Abstract

*Juniperus*, a genus shaped by long-term climatic and geological processes, thrives in Yunnan but its community structure and future habitat dynamics remain poorly understood. We investigated *Juniperus* community composition, phylogenetic diversity, population structure, and projected suitable future habitats using MaxEnt modeling. Seven distinct community types were identified, all dominated by *Juniperus* species. They were characterized by low species richness, strong dominance patterns, and uneven species distribution. Most communities displayed a multi-layered vertical structure, except Type 6. Phylogenetic analyses revealed overdispersion (NRI < 0) in most communities, indicating niche differentiation or competitive exclusion as the primary assembly process, while Type 2 (NTI > 0) comprised more closely related species, potentially due to external disturbance. Population structures varied: *J. pingii* var. *wilsonii* exhibited an inverse J-shaped DBH distribution, *J. formosana* and *J. squamata* had unimodal patterns, and other species showed multimodal distributions. Climate projections forecast a 4.5–41.9% loss of suitable habitat, with current nature reserves covering only 13.6–35.1% of stable refugia, highlighting the need for targeted conservation. These findings provide an essential basis for the conservation, afforestation, and restoration of *Juniperus*-dominated ecosystems in Yunnan.

## 1. Introduction

*Juniperus* (Cupressaceae) is an important component of arid and semi-arid ecosystems in the Northern Hemisphere and is mainly distributed in temperate, semi-arid habitats [1,2,3]. Fossil records (Figure S1) indicate that the genus *Juniperus* originated in Southern Eurasia during the Eocene [4]. And following numerous geological and historical events, it evolved into its current distribution across North America, Asia, Europe, and Africa [3]. Yunnan Province, which covers only 4.1% of the total area of China, is home to 12 species of *Juniperus*, accounting for 17% of the species on the global scale, and 52% of the species in China [5], making the Hengduan Mountains area the primary center of modern distribution and diversification for *Juniperus* species in China [6,7]. Hence, Yunnan is an optimal site for conducting research on this genus.

Some *Juniperus* species have been studied by researchers from various aspects, including the ecology of *Juniperus occidentalis* Hook in the Western United States [8], the communities of *J*. *polycarpos* K.Koch in the Himalayan region of India [9], the populations of *J*. *communis* L. in the Iberian peninsula [10], the genetics of *Juniperus* worldwide [11,12], the habitat suitability of *Juniperus* in Southern Iran [13], and a few studies of *Juniperus* in China, such as the population structure, spatial patterns and associations of *J*. *saltuaria* Rehder & E. H. Wilson on the Southeastern Tibetan Plateau [14], the phylogenetic relationships of five genera of Cupressaceae [15], and the extinction risk of three *Juniperus* species under climate change in Southwestern China [16]. Evidently, information on other *Juniperus* species and their populations, communities and conservation status in China is very scarce.

In this paper, seven *Juniperus* species in Yunnan are selected for study, including *J*. *pingii* W. C. Cheng ex Ferré, *J. formosana* Hayata, *J. coxii* A.B.Jacks., *J. squamata* D.Don, *J. saltuaria*, *J. pingii* var. *wilsonii* (Rehder) Silba and *J. indica* Bertol. Each of them is a primary dominant in its forest community. The conservation status of these species is of significant concern, particularly for *J. pingii* and *J. coxii*, which are listed as Vulnerable and Near-threatened on the IUCN Red List, respectively; *J. coxii* is additionally assessed as Near-threatened on the Threatened Species List of China’s Higher Plants [17,18]. These *Juniperus* species (except *J. coxii*, *J. squamata* and *J. indica*) are endemic to China. *Juniperus* species have high medicinal and economical values, as they contain compounds such as flavonoids [19], lignin [20], and diterpenes [21], as well as volatile oil components [22], which have anti-inflammatory and antibacterial effects. Due to their ability to tolerate cold and drought, and their longevity, purifying properties and aromatic qualities, they are often used for architectural construction, making furniture, and landscaping, and are a powerful symbol of protection, healing and spiritual resilience across cultures and time periods. They hold significant economical and ecological as well as cultural importance. Studying their community characteristics and population structure is an important task for effectively protecting the *Juniperus* species in the wild and promoting plant diversity.

In the past, ecologists used species diversity indicators to characterize the richness of species within a community, however, these indicators did not take into account the phylogenetic relationships among species [23,24,25,26]. The current species composition within a community result from the interplay between evolutionary and ecological processes, and analyzing interspecies phylogenetic relationships could illuminate the historical processes that shaped contemporary communities [27]. Generally, species with closer phylogenetic relationships tend to have more similar ecological niches [28]. If habitat filtering is dominant during community assembly, the same habitat will select for species with similar adaptive abilities and closer phylogenetic relationships; conversely, species with similar ecological niches cannot coexist in the same habitat due to competitive exclusion, leading to greater divergence in the phylogenetic relationships of species [27,29]. Phylogenetic information can contribute to understanding how communities assemble and the forces that influence their dynamics, diversity and ecosystem function [30]. To quantify the phylogenetic structure within communities, Webb introduced the Net Relatedness Index (NRI) and the Nearest Taxon Index (NTI) [27]. NRI assesses the degree of phylogenetic clustering or overdispersion at the whole-community level, whereas NTI focuses on the phylogenetic structure of terminal branches [27]. Analyzing the phylogenetic diversity of the communities dominated by *Juniperus* species can help to understand their community characteristics.

Over the past three decades, compelling evidence has indicated that the continuously escalating concentrations of greenhouse gases in the atmosphere have triggered changes in the global climate. Due to climate change disrupting specific environmental conditions, species may no longer be able to adapt to their original climate niche [31]. Driven by global warming, montane plants are migrating upslope, yet the restricted space of alpine zones imposes a severe risk of habitat loss [32]. Consequently, simulating the potential distribution pattern of a species based on its environmental ecological requirements could disclose the areas that may encounter high extinction rates and facilitate the assessment of threats to species survival [33,34,35,36,37]. Therefore, it is necessary to conduct studies on predicting the potential distribution areas of these *Juniperus* species to inform conservation management and promote sustainable utilization.

Ecological niche modeling (ENM) is widely employed to project species distributions by quantifying the relationships between occurrence records and environmental predictors, thereby delineating the realized niche and habitat suitability [38]. Currently, the most commonly employed ecological niche models include the Genetic Algorithm for Rule-set Prediction (GARP) [39], Random Forests (RFs) [40], and MaxEnt [41]. The Maximum Entropy Model (MaxEnt) algorithm is regarded as the currently best-performing ecological niche model due to its features of faster computing speed and more accurate results [41,42,43]. Previous research has applied the MaxEnt model to predict the potential suitable habitats of individual *Juniperus* species in various parts of the world. For example, Pérez-Suárez et al. [44] modeled the distributions of *J. jaliscana*, *J. monticola*, and *J. pinchotii* in Mexico; Wang et al. [45] examined *J. tibetica* in China; and Turkmenoglu et al. [46] assessed *J. drupacea* in Türkiye. These studies have provided valuable insights into species-specific habitat requirements and responses to climate change. However, no research to date has conducted a region-wide, multi-species analysis of *Juniperus* in Yunnan, a biodiversity hotspot with unique climatic and topographic conditions. This study fills that gap by systematically modeling the potential suitable habitats of multiple *Juniperus* species in Yunnan under current and future climate scenarios, offering a comprehensive perspective that can inform both regional conservation planning and broader biogeographic understanding of the genus.

Understanding the plant community, population and regeneration characteristics, phylogenetic diversity, and future suitable habitat distribution of *Juniperus* is essential for gaining insights into their plant community and population dynamics and provides a reference for re-forestation and conservation as well as site restoration planning. To date, the communities containing *Juniperus* as the 1st dominant have not been systematically studied in Yunnan. In this study, we aim to answer the following questions: 1. What are the community characteristics dominated by *Juniperus* species in Yunnan? 2. What is the phylogenetic diversity of these communities? 3. What are the population structures and regeneration status of these *Juniperus* species? 4. What are the potential distribution areas for these *Juniperus* species in the future?

## 2. Materials and Methods

### 2.1. Study Area

**Figure 1:**
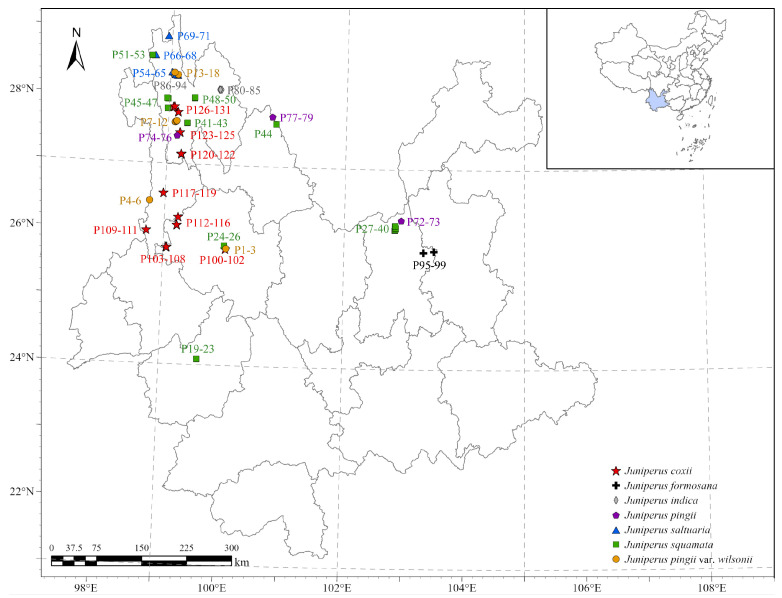
Distribution of plots of plant communities containing *Juniperus* species as the 1st dominant.

We conducted extensive surveys in all accessible areas within Yunnan Province to identify plant communities where a species of *Juniperus* was the primary dominant species. We established a total of 131 plots across 6 prefectures and 14 counties in the central-eastern, western, and northwestern areas of Yunnan (Figure 1). Detailed plot locations and environmental characteristics are provided in Supplementary Table S1. The summer climate of the study areas is mainly influenced by the Indian Ocean monsoon. For these plot sites, the annual average temperature ranges from 0.1 °C to 19.3 °C. The average temperature of the warmest month (July) ranges from 4.8 °C to 25.3 °C, and the average temperature of the coldest month (January) ranges from −7.4 °C to 11.4 °C. The annual precipitation ranges from 526 to 1228 mm, the actual evapotranspiration ranges from 343 mm to 836 mm, and the moisture index ranges from 0.8 to 1. These data on the plot sites were extrapolated from the observed data covering 50 years (1950–2000) from local climatological stations.

### 2.2. Community and Population Characteristics

#### 2.2.1. Field Investigation

In 2020 and 2023, we conducted surveys in all patches containing *Juniperus* species across Yunnan. A total of 131 sampling plots dominated by *Juniperus* species were established. Plot sizes varied from 10 m × 20 m to 20 m × 20 m, selected based on the minimum area required to capture maximum species richness, patch size, and topographic constraints. Within each plot, all woody individuals with a height ≥ 1.3 m were identified to species level, numbered, marked, and measured for diameter at breast height (DBH) and full height. Additionally, general information about each relevant plot was documented, including elevation, slope position, slope aspect, slope inclination, and disturbance history.

#### 2.2.2. Forest Structure

Woody individuals in the overstory (height ≥ 1.3 m) were classified into two categories based on their vertical position and height: arborous layer (height ≥ 5 m) and shrub layer (1.3 m ≤ height < 5 m tall) [47]. The arborous layer included emergent (height > 20 m), canopy (10 m ≤ height ≤ 20 m), and subcanopy (5 m ≤ height < 10 m) sublayers. Woody individuals in the understory (height < 1.3 m) were divided into two categories based on size [48,49]: (1) juveniles (5 cm < height ≤ 60 cm), representing young non-reproductive individuals beyond the seedling stage, and (2) saplings (60 cm < height ≤ 130 cm), referring to taller juvenile woody plants not yet mature. Within these two categories for the understory, each individual was identified to species, counted, and measured for height and percentage of coverage. For herbaceous species, five 1 m × 1 m subplots were established at the four corners and the center of each plot. Within each subplot, we identified all herbaceous species, estimated their percent coverage representing the proportion of the vertical projection area of a focal species to the total area of the understory of a subplot, and recorded the number of individuals per species. Sprouts (clonal ramets) and their main stems were treated as a single individual.

#### 2.2.3. Community Classification

Forest classification was performed based on species importance values (which integrate relative density and relative basal area) using Euclidean (Pythagorean) distance and McQuitty’s linkage method in PC-ORD [50]. A dendrogram was constructed to group plant communities according to similarities in community species composition and structure. To determine the optimal community classification, we plotted the fusion-level diagram of the dendrogram to locate the cutting height, then selected the best number of clusters by maximizing the Mantel statistic [51], as shown in Figure S2.

#### 2.2.4. Species Diversity and Floristic Features

For each vegetation plot, we calculated species richness, Shannon–Wiener diversity index, Pielou’s evenness index and Simpson index. The Shannon-Wiener index provides a comprehensive measure of species diversity by considering both species richness and species uniformity [52]. Pielou’s evenness index measures the uniformity of the distribution of individual species within a community, and the Simpson diversity index quantifies the species diversity within a community [53]. These indices are widely used in ecological studies to quantify biodiversity and community composition [47,48,54]. To compare diversity among the seven identified community types, we calculated the mean and standard deviation of each index across all plots within the same community type. This approach is standard in community ecology, as each community type is represented by multiple plots, and the mean index value provides a representative measure for that type. Differences among community types were analyzed by the nonparametric Kruskal–Wallis all-pairwise comparisons test (*p* < 0.05), using R version 4.3.2 [55].

For floristic features, we classified the geographic distribution types of seed plant families and genera by Wu’s system [56,57].

#### 2.2.5. Phylogenetic Analysis

To construct a phylogenetic tree of *Juniperus* species in Yunnan Province, we compiled a species list of all woody species ≥ 1.3 m in height observed in the surveyed plots, and verified the list with the WFO Plant List (http://wfoplantlist.org/taxon, accessed on 5 March 2025) using the “plantlist” package [58]. According to the list, we generated a phylogenetic tree based on the time-calibrated phylogeny of Smith and Brown [59] using the package ‘V.PhyloMaker’ [60,61].

To analyze the differences in phylogenetic indices among the plant community types of *Juniperus* species in Yunnan Province, we used the “picante” package [62] to calculate the NRI and NTI values for all species in each community group. Subsequently, we conducted descriptive statistical analysis based on the NRI and NTI values and created box plots. All calculations were performed in R version 4.3.2 [55].

#### 2.2.6. *Regeneration Analysis*

The juveniles and saplings of *Juniperus* were predominantly observed in specific microhabitats, which collectively constitute only a small fraction of the total plot area, such as under canopy, canopy gaps, forest edges, road side and rock crevices.

To reveal the regeneration potential of *Juniperus* communities, we measured the height, density and distribution sites of the juveniles and saplings. And then we employed Generalized Linear Models (GLM) to separately plot the fitted curves of height–density and microhabitat–density for juveniles and saplings in R version 4.3.2 [55]. Since microhabitats are categorical variables, in this study, different levels of disturbance intensity were assigned to microhabitats: 1, Under canopy; 2, Canopy gaps; 3, Forest edges; 4, Road side; 5, Rock crevices.

### 2.3. Future Potential Habitats Under Climate Change

#### 2.3.1. Occurrence Data

To study the future distribution patterns of the seven *Juniperus* species, we compiled distribution data for each species and used the MaxEnt model to individually predict the future suitable habitats for each one. Distribution data for *Juniperus* species across East Asia were compiled from the Chinese Virtual Herbarium (CVH; http://www.cvh.ac.cn, accessed on 18 March 2025), from various printed sources, such as e-floras of relevant countries in East Asia, monographs and journal articles and our field investigation. To reduce sampling bias and avoid model overfitting, we used the “raster” package to filter the distribution data with a spatial resolution of 2.5 arc-minutes. This process resulted in a final dataset of 1364 coordinate records.

#### 2.3.2. Climate Data

All climate data were downloaded from the website: WorldClim (http://worldclim.org, accessed on 20 March 2025), with a spatial resolution of 2.5 arc-minutes, including 19 environmental variables. Current climate data were based on global meteorological records from 1970–2000, while future climate (average for 2080–2100) projections used data from the BCC-CSM2-MR atmospheric circulation model under the CMIP6 framework, specifically under SSP126 and SSP585 scenarios provided by the National Climate Center [63]. To account for potential dispersal into suitable habitats, we defined a study area much larger than the species’ current distribution range.

#### 2.3.3. Climate Variable Screening

Strong covariance among bioclimatic variables will affect the accuracy of predictive distribution models [64]. Reducing multicollinearity among predictor variables is crucial for building reliable predictive distribution models. Therefore, we screened 19 climate variables by the “SDMtune” package [65], and ultimately retained six variables for subsequent *Juniperus* MaxEnt distribution modeling. These variables are: bio2 (Mean Diurnal Range), bio3 (Isothermality), bio6 (Min Temperature of Coldest Month), bio7 (Temperature Annual Range), bio15 (Precipitation Seasonality), and bio17 (Precipitation of Driest Quarter).

#### 2.3.4. Parameter Optimization

To avoid overfitting and maximize the predictive capability of the model, we optimized the parameter settings for MaxEnt. We calculated the Akaike Information Criterion (AICc) using the “ENMeval” package [66,67] to assess the performance of the MaxEnt model under different combinations of environmental variables and model parameters. Additionally, we employed 10-fold cross-validation to ensure the robustness of the model.

The receiver operating characteristic (ROC) curve was plotted to assess the model’s goodness of fit, with the Area Under the Curve (AUC) representing model performance. The AUC ranges from 0 to 1, with higher values indicating greater reliability of the predictions. Specifically, AUC values are interpreted as follows: poor (<0.7), fair (0.7–0.8), good (0.8–0.9), and excellent (>0.9). All calculations were performed in R version 4.3.2 [55].

#### 2.3.5. Future Suitable Habitat Prediction

In MaxEnt version 3.4.4, we imported the filtered coordinate records and environmental factors, setting the optimal run configuration for the *Juniperus* species MaxEnt prediction model based on the model with the lowest ΔAICc value [68]. We employed the bootstrap method, running the model 10 times to obtain more robust modeling results. The average of these ten runs was taken as the output of the model. Subsequently, we imported the results into ArcGIS Pro 2.5 for reclassification. We selected thresholds based on maximum training sensitivity plus specificity logistic threshold to distinguish suitable and unsuitable areas within the model, which could effectively balance the sensitivity and specificity of the model, thereby enhancing the model’s predictive ability [69]. The suitability was categorized into different levels: 0-threshold, threshold−0.4, 0.4–0.6, and 0.6–1. We performed statistical analysis on each class layer to determine the area of suitable habitats for *Juniperus* species, and then identified the main environmental factors influencing their geographic distribution based on the relative contribution rates of each climatic factor from the simulation results.

## 3. Results

### 3.1. Community Characteristics

#### 3.1.1. Community Types and Stratification

**Figure 2:**
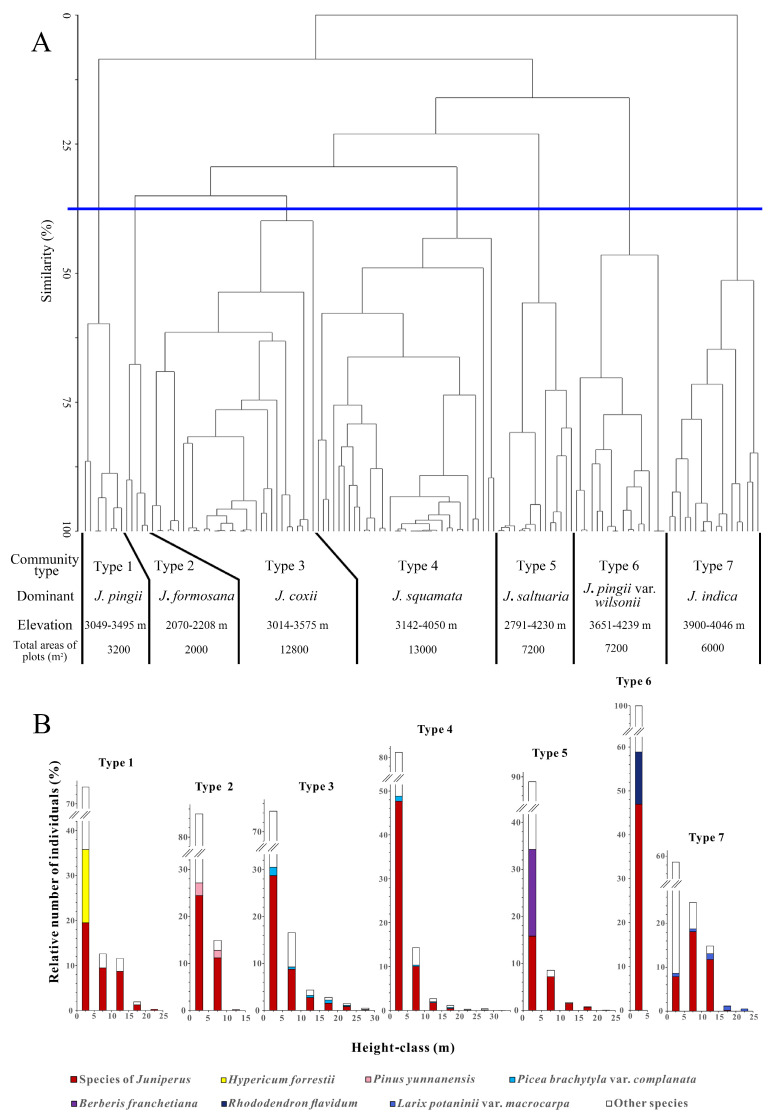
(**A**) Dendrogram of floristic similarity for 131 plots; (**B**) The frequency distribution in height-classes of woody species (height ≥ 1.3 m) of each community type. Type 1: *J. pingii* evergreen coniferous forest; Type 2: *J. formosana* evergreen coniferous forest; Type 3: *J. coxii* evergreen coniferous forest; Type 4: *J. squamata* evergreen coniferous forest; Type 5: *J. saltuaria* evergreen coniferous forest; Type 6: *J. pingii* var. *wilsonii* evergreen coniferous shrub community; Type 7: *J. indica* evergreen coniferous forest. *J.* = *Juniperus*.

The results of the fusion-level diagram and the Mantel statistic, which classified 131 vegetation plots into seven distinct plant communities, are presented in Figure S2. Combining the Floristic and structural similarity cluster analysis, the 32.5% similarity threshold best matched our field-based observations of vegetation composition, structure, and habitat differentiation (Figure 2A). Each of the seven distinct community types corresponds well with observable ecological patterns and dominant species groupings noted during field surveys. While alternative thresholds were explored, the 32.5% level offered the most ecologically meaningful and interpretable grouping, balancing both statistical differentiation and biological realism. The vertical stratification of woody plants (height ≥ 1.3 m) in each community type is illustrated in Figure 2B.

Community Type 1: *J. pingii* evergreen coniferous forest, distributed in the mountainous upper slopes, outcrop-rich limestone habitats, and steep slopes at high elevations between 3049–3495 m in central Yunnan (Dongchuan County) and northwestern Yunnan (Weixi and Ninglang Counties). The canopy and subcanopy were dominated by *J. pingii* (RIV = 75.47%), and there were a few trees reaching heights of 20–22 m. A few individuals of *Pinus armandi*, and *Quercus longispica* were present, along with *Quercus senescens* in the canopy and subcanopy. In the shrub layer, the dominants were *J. pingii* (RIV = 26.71%) and *Hypericum forrestii* (RIV = 30.29%), accompanied by *Elsholtzia fruticosa* and *Berberis franchetiana*. In the understory, there were herbaceous *Fragaria vesca*, *Hydrocotyle nepalensis* and *Potentilla chinensis*, etc.

Community Type 2: *J. formosana* evergreen coniferous forest, found on the upper slopes along roadsides and limestone habitats near villages at mid-high elevations of 2070–2208 m near the mountain summit in central Yunnan (Xundian County). Only a few *Pinus yunnanensis* trees exceeding 10 m in height were found in the forest. Numerous individuals of *J. formosana* (RIV = 65.17%) were concentrated in the subcanopy layer and co-dominated with *Pinus yunnanensis* (RIV = 19.02%). *J. formosana* (RIV = 26.06%) also dominated the shrub layer, along with *Berberis pruinosa*, *Viburnum congestum*, and *Pyracantha fortuneana*. The common herbaceous species was *Heteropogon contortus* in the understory.

Community Type 3: *J. coxii* evergreen coniferous forest, located in gullies near mountain summits, on mountain slopes and steep slopes, in limestone habitats near hilltops at high elevations of 3014–3575 m in northwestern Yunnan (Deqin and Weixi Counties), western Yunnan (Fugong County, Lushui City), and central Yunnan (Yangbi and Yunlong Counties). The maximum height of *J. coxii* reached 27 m. *J. coxii* (RIV = 56.17%) dominated the canopy and subcanopy. A few trees of *Tsuga dumosa*, *Picea brachytyla* var. *complanata*, and *Abies georgei* were found in the canopy and subcanopy, accompanied by *Rhododendron irroratum* and *Rhododendron aganniphum*. In the shrub layer, *J. coxii* (RIV = 45.1%) was dominant. In the understory, herbs of *Rubus fockeanus* and *Argentina lineata* were common.

Community Type 4: *J. squamata* evergreen coniferous forest, distributed in outcrop-rich limestone habitats, on mountain slopes and steep slopes near mountain summits at elevations of 3142–4050 m in northwestern Yunnan (Ninglang, Weixi and Deqin Counties), central Yunnan (Dali City, Luquan County), and southwestern Yunnan (Yongde County). A few trees of *Abies georgei*, and *Picea brachytyla* var. *complanata* reached the emergent layer. *J. squamata* dominated the canopy and subcanopy (RIV = 57.66%), along with *Picea brachytyla* var. *complanata*, *Abies georgei*, and *J. coxii*, etc. In the shrub layer, *J. squamata* (RIV = 54.68%) was dominant, accompanied by *Rhododendron sphaeroblastum* var. *wumengense*, *Rhododendron wardii* and *Berberis dictyophylla*, etc. In the understory, various herbaceous *Fragaria vesca*, *Argentina phanerophlebia*, *Fragaria nilgerrensis*, etc. were found.

Community Type 5: *J. saltuaria* evergreen coniferous forest, found on mountain slopes, steep slopes and in limestone habitats at high elevations of 2791–4230 m in northwestern Yunnan (Deqin County). The canopy and subcanopy were dominated by *J. saltuaria* (RIV = 85.7%), with *Larix potaninii* var. *australis* and *Abies georgei* as associated species. In the shrub layer, *J. saltuaria* (IV = 20.5%) and *Berberis franchetiana* (RIV = 12.3%) were co-dominants, accompanied by *Ostryopsis nobilis*, *Rhododendron phaeochrysum*, and *Berberis wilsoniae*. In the understory, herbaceous *Primula polyneura* was a dominant.

Community Type 6: *J. pingii* var. *wilsonii* evergreen coniferous shrub community, found on mountain slopes, outcrop-rich sites and steep slopes at high elevations of 3651–4239 m in northwestern Yunnan (Deqin and Weixi Counties), central Yunnan (Dali City), and western Yunnan (Lushui City). Due to the biological traits of *J. pingii* var. *wilsonii*, this community lacked canopy and subcanopy. The shrub layer was absolutely dominated by *J. pingii* var. *wilsonii* (RIV = 48.64%), accompanied by *Rhododendron flavidum*, *Rhododendron rupicola* var. *chryseum*, *Dasiphora fruticosa*, and *Dasiphora glabra*, etc. In the understory, there were herbs of *Viola biflora*, *Bistorta vivipara* and *Potentilla chinensis*, etc.

Community Type 7: *J. indica* evergreen coniferous forest, occurring along riversides, and on mountain slopes at high elevations of 3900–4046 m in northwestern Yunnan (Deqin County, Shangri-La City). The canopy and subcanopy were dominated by *J. indica* (RIV = 72.73%), accompanied by *Larix potaninii* var. *australis*, *Abies georgei*, and *Abies georgei* var. *smithii*. The shrub layer exhibited co-dominance of *J. indica* (RIV = 17.09%) and *Rhododendron phaeochrysum* (RIV = 12.99%), associates including *Quercus pannosa* and *Spiraea schneideriana*, *Rhododendron rubiginosum*, and *Lonicera trichosantha*, etc. The understory was dominated by herbaceous *Primula polyneura*.

**Figure 3:**
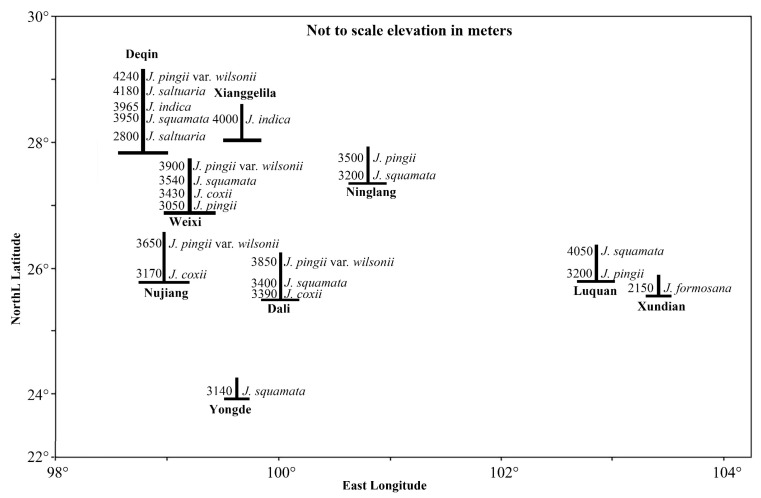
The spatial distribution pattern of representative communities containing *Juniperus* species as a dominant in Yunnan. *J.* = *Juniperus*.

Figure 3 shows the distribution pattern of plant communities dominated by *Juniperus* species along latitudinal, longitudinal, and elevational gradients in Yunnan. The species richness of *Juniperus* in the northwestern area was higher than the other areas of Yunnan. All the *Juniperus* communities were distributed in locations close to the mountain summits.

**Figure 4:**
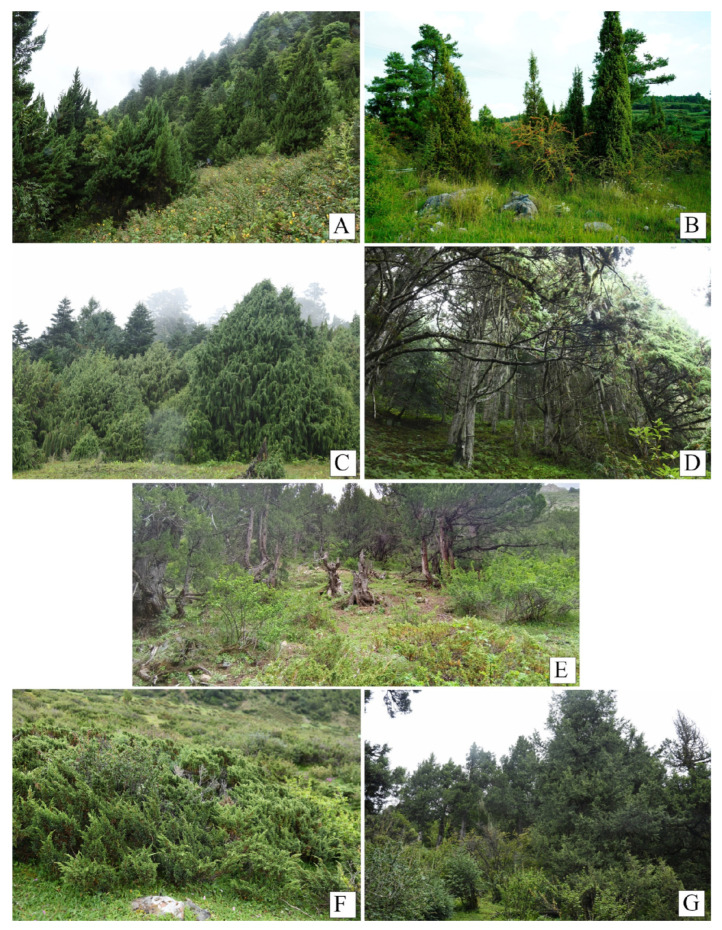
Representative communities and habitats of *Juniperus* species in Yunnan. (**A**) *J. pingii* evergreen coniferous forest (Type 1) in Weixi county, 3050 m a.s.l. (above sea level) (P75) (**B**) *J. formosana* evergreen coniferous forest (Type 2) in Xundian county, 2208 m a.s.l. (P97); (**C**) *J. coxii* evergreen coniferous forest (Type 3) in Weixi county, 3475 m a.s.l. (P131); (**D**) *J. squamata* evergreen coniferous forest (Type 4) in Weixi county, 3530 m (P48); (**E**) *J. saltuaria* evergreen coniferous forest (Type 5) in Deqin county, 4225 m a.s.l. (P70); (**F**) *J. pingii* var. *wilsonii* evergreen coniferous shrub community (Type 6) in Deqin county, 4239 m a.s.l. (P17); (**G**) *J. indica* evergreen coniferous forest (Type 7) in Xianggelila county, 4020 m a.s.l. (P85).

Figure 4A–G displays the representative communities and habitats of the seven *Juniperus* species. It is clear that these seven forest types are highly heterogeneous, characterized by distinct species compositions, dynamics, and complex structures. Among these community types, all except Type 6 (*J. pingii* var. *wilsonii* evergreen coniferous shrub community) exhibit multiple layers of stratification, comprising the arborous layer, shrub layer, and understory.

#### 3.1.2. Species Diversity and Phylogenetic Diversity

In all the *Juniperus* communities, we recorded a total of 498 plant species, belonging to 81 families and 234 genera. Among them, there are 2 families, 7 genera, and 18 species of gymnosperms; 29 families, 215 genera, and 463 species of angiosperms; and 10 families, 12 genera, and 17 species of ferns. Among all the seed plants, approximately 52.3% of the families and 23.6% of the genera represented tropical elements, while 43.2% of the families and 61.8% of the genera had temperate elements. The higher proportion of temperate elements at the genus level indicates that the species composition in the *Juniperus* communities is primarily temperate (Supplementary Table S2).

**Figure 5:**
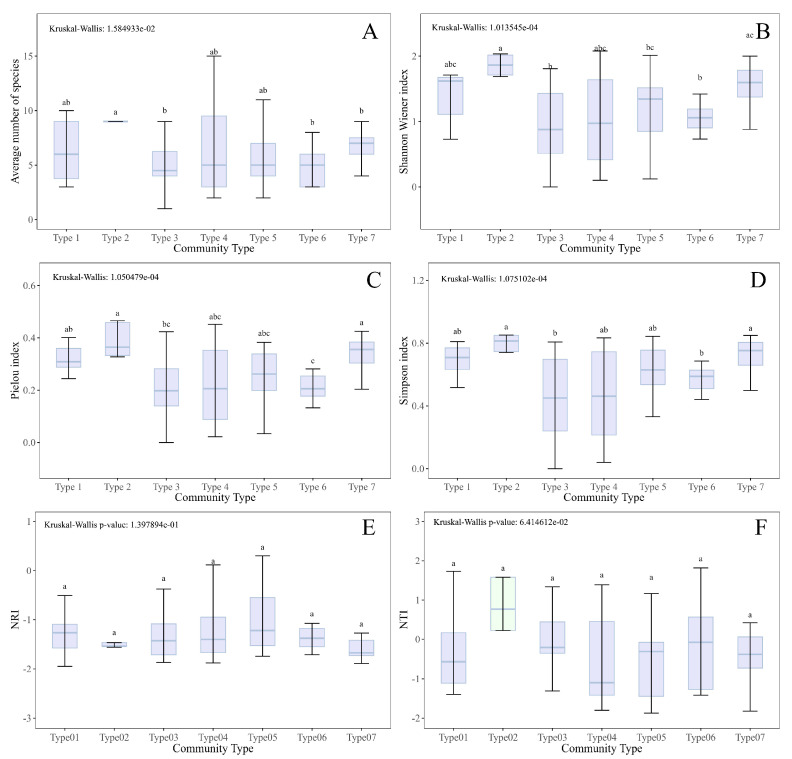
(**A**–**F**): Woody species (height ≥ 1.3 m) richness, diversity indices, and phylogenetic diversity indices of each community type, calculated as the mean ± standard deviation of all vegetation plots within that community type. (**A**–**D**): Species richness, Shannon–Wiener index, and Pielou’s evenness index and Simpson index of each community type. (**E**,**F**): Species phylogenetic diversity NRI (Net Relatedness Index) and NTI (Nearest Taxon Index) values of each community type. Type 1: *J. pingii* evergreen coniferous forest; Type 2: *J. formosana* evergreen coniferous forest; Type 3: *J. coxii* evergreen coniferous forest; Type 4: *J. squamata* evergreen coniferous forest; Type 5: *J. saltuaria* evergreen coniferous forest; Type 6: *J. pingii* var. *wilsonii* evergreen coniferous shrub community; Type 7: *J. indica* evergreen coniferous forest. Communities sharing different letters differ significantly, and sharing the same letters denotes that they do not differ significantly by the nonparametric Kruskal–Wallis all-pairwise comparisons test (*p* < 0.05). Bar: Standard deviation.

The species richness and diversity indices of woody plants (height ≥ 1.3 m) in each community type are shown in Figure 5A–D. The average woody species richness of plots across the different community types ranged from four to nine species. The average species richness in Type 2 (*J. formosana* evergreen coniferous forest) was significantly higher than those in Type 3 (*J. coxii* evergreen coniferous forest), Type 6 (*J. pingii* var. *wilsonii* evergreen coniferous shrub community) and Type 7 (*J. indica* evergreen coniferous forest), while no significant differences were found among the other community types (Figure 5A). The Shannon–Wiener diversity index was between 0.91 and 1.86, the Simpson diversity index ranged from 0.45 to 0.8, and the Pielou evenness index ranged from 0.2 to 0.39 among all the seven community types (Figure 5B–D). In general, Type 2 (*J. formosana* evergreen coniferous forest) and Type 7 (*J. indica* evergreen coniferous forest) had significantly higher diversity indices than Type 3 (*J. coxii* evergreen coniferous forest) and Type 6 (*J. pingii* var. *wilsonii* evergreen coniferous shrub community) (Figure 5B–D).

The species composition with relative importance values of each community type is shown in Tables S3–S5.

The phylogenetic diversity and relationship analysis of species across the seven community types is illustrated in Figure 5E,F. The NRI (Net Relatedness Index) values for all community types are less than 0. The NTI (Nearest Taxon Index) values for all the community types were also less than 0, except for that of Type 2 (*J. formosana* evergreen coniferous forest), which was above 0. Overall, although there are certain differences in the NRI and NTI values among different community types, these differences have not reached the level of statistical significance (Figure 5E,F).

### 3.2. Population Structure

#### DBH-Class Structure and Regeneration

**Figure 6:**
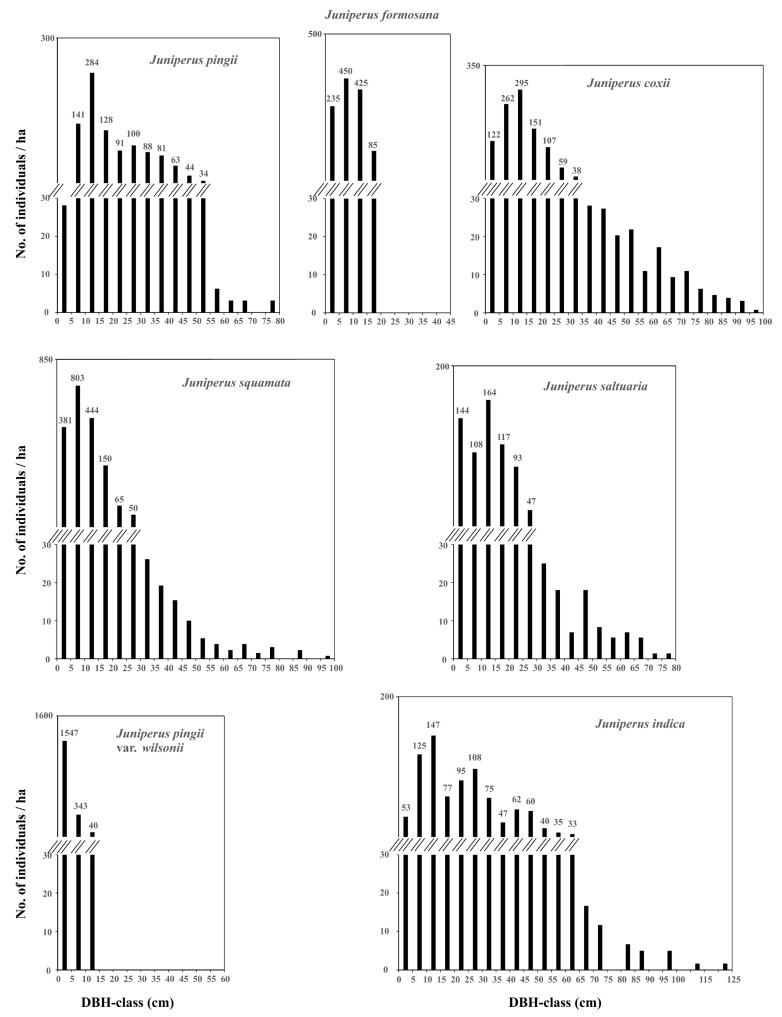
The frequency distribution in DBH-classes of dominant species in each community type.

The DBH-class frequency distribution of individuals of *Juniperus* species in each community type is illustrated in Figure 6. Among these, *J. pingii* var. *wilsonii* in community Type 6 exhibited an inverse J-shaped distribution in a small range of 0–15 cm DBHs, while *J. formosana* in community Type 2 displayed a unimodal distribution with a peak at 5–10 cm DBHs within a small range of 0–20 cm DBHs. *J. squamata* in community Type 4 also showed a unimodal distribution with a peak at 5–10 cm DBHs within a large range of 0–100 cm DBHs. Each of the other four *Juniperus* species in its own community type exhibited a multimodal distribution within ranges of 0–80 cm, 0–100 cm, and 0–125 cm DBHs. In general, there were more individuals between 5–15 cm DBHs than those in the other DBH-classes for the *Juniperus* species except *J. pingii* var. *wilsonii*.

The density of juveniles and saplings of each *Juniperus* species in various micro-habitats is shown in Figure S3. The juveniles and saplings of *Juniperus* species were mainly found in canopy gaps and roadsides. Some juveniles of *J. squamata*, *J. pingii* var. *wilsonii* and *J. indica* were found in rock crevices but only a small number of *Juniperus* species juveniles were found under the canopy and at forest edges. No juveniles of *J. formosana* were found. Based on the GLM analysis of juveniles and saplings, we found that the number of juveniles and saplings within all community types was negatively correlated with their height (Figure S4). In the *J. pingii* and *J. saltuaria* communities, the number of juveniles and saplings was negatively correlated with the intensity of habitat disturbance, while in the other four community types, it was positively correlated. Established saplings (with heights of 90–130 cm) were very rare for *J. squamata* and none were found for the other six *Juniperus* species (Figure S3).

### 3.3. Predicted Habitats Under Climate Change

#### 3.3.1. Potential Distribution Under Current Climate

Utilizing the MaxEnt model, we conducted 10 repetitions for each species. The mean AUC values for all *Juniperus* species exceeded 0.9, indicating robust model performance for predicting their distributions. As previous studies have suggested, an AUC value above 0.90 is considered “excellent”.

We predicted the potential suitable habitats for the seven *Juniperus* species as expressed by suitability. Our model demonstrated excellent performance (Supplementary Table S6). Among the six environmental factors, bio3 (Isothermality), bio6 (Min Temperature of Coldest Month) and bio7 (Temperature Annual Range) emerged as the most significant variables determining the potential distribution of these species (Table S6).

Based on current distribution records, we predicted the potential suitable habitats in East Asia for the seven *Juniperus* species under current climatic conditions, as shown in Supplementary Figure S5A–G. The habitats of each *Juniperus* species are mainly located between latitudes 20° N and 40° N. *J. formosana* has the largest potential suitable habitats among the seven species. Its areas with high suitability (0.6–1) are distributed in the Wuyi Mountains, Wushan-Wuling Mountains, and Qinling-Daba Mountains; the areas with medium suitability (0.4–0.6), which are surrounded by the areas with low suitability (0.2–0.4), are widespread in southeastern China. For the other six *Juniperus* species, although the areas of potential habitats vary, the distribution patterns of these species are similar. The areas with high suitability are predominantly located in the Hengduan Mountains and the eastern Himalayas, such as the northwest of Yunnan, the southwest of Sichuan, the south and southeast of Tibet.

#### 3.3.2. Potential Distribution Under Future Climate

**Figure 7:**
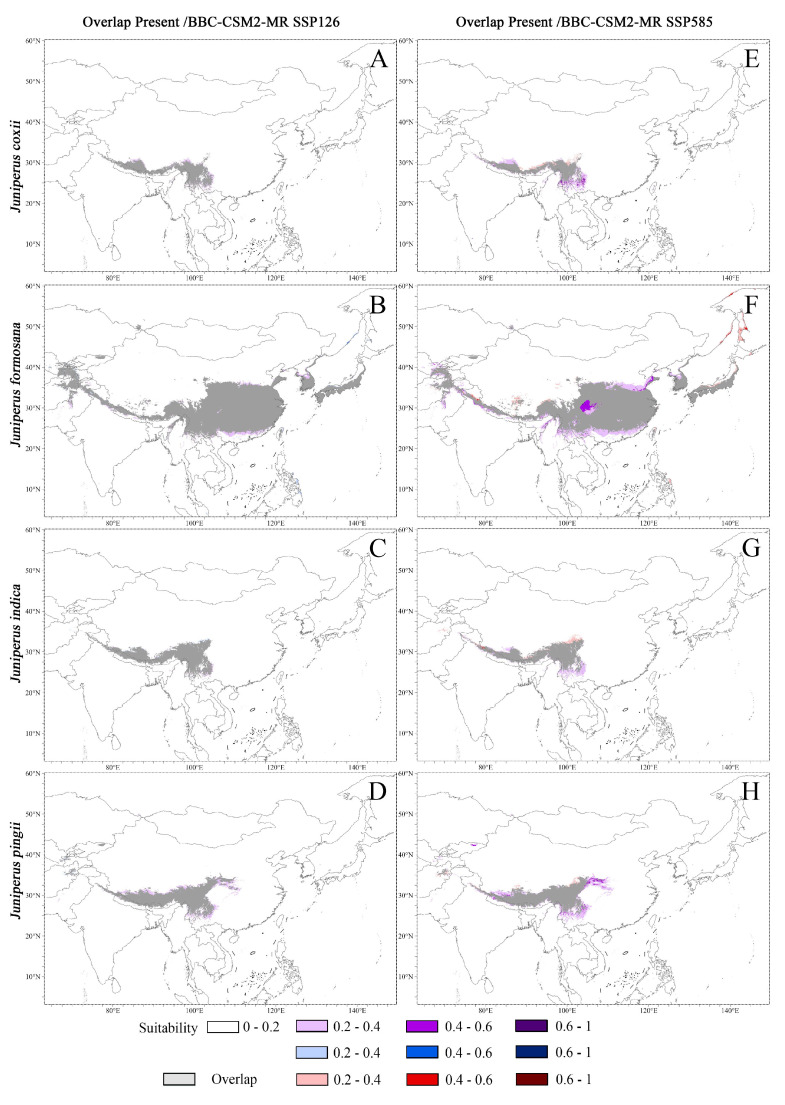

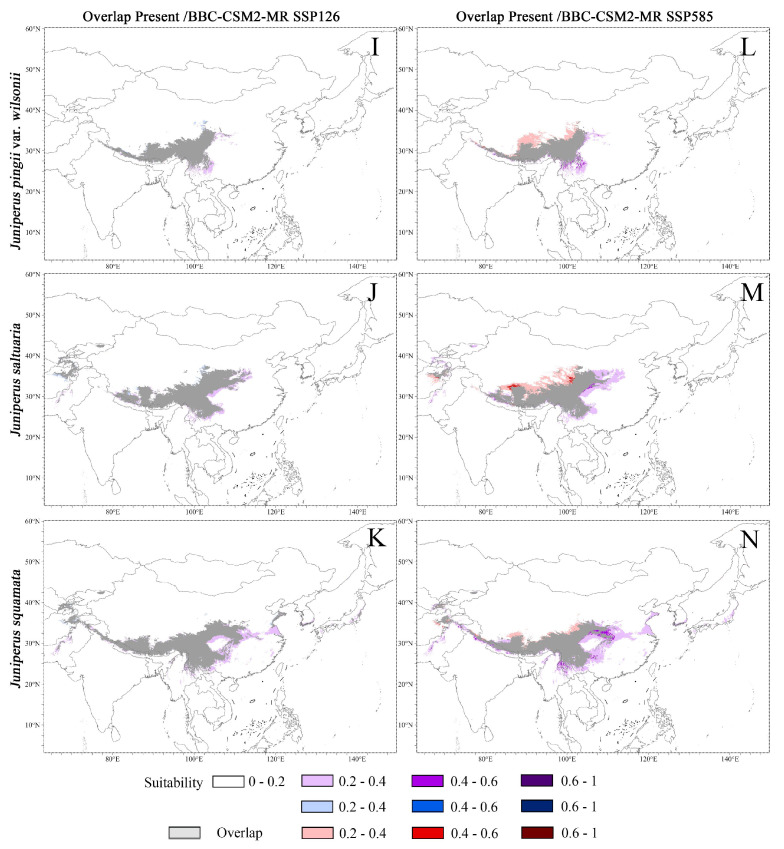
The overlap areas of potential habitats under two climatic scenarios in the future (2100). (**A**–**D**,**I**–**K**): Overlap areas of the 2100-BCC-CSM2-MR SSP126 and the present. (**E**–**H**,**L**–**N**): Overlap areas of the 2100-BCC-CSM2-MR SSP585 and the present.

We used scenarios 2100-BCC-CSM2-MR SSP126 and 2100-BCC-CSM2-MR SSP585 to model potential climatic habitats of the seven *Juniperus* species in the future (2100) (Supplementary Table S7, Figure S6 Part1 A–H and Figure S6 Part2 I–N). The overlap areas (gray color) of potential habitats under the present climate and two climatic scenarios in the future (2100) indicate that the total area of the potential climatic habitats of all *Juniperus* species would be decreased in the future compared to the present (Figure 7A–N).

Under the 2100-BCC-CSM2-MR SSP126 scenario, the areas of potential habitats for all *Juniperus* species would be somewhat diminished, and most of the potential habitats for the year 2100 would be included within the current potential habitats (the overlap areas in gray in Figure 7A–D,I–K). Although the species with the largest area loss of potential habitats with a suitability of 0.2–1 would be *J. squamata*, the species with the largest are loss of high potential habitats with a suitability of 0.6–1 would be *J. formosana* (Table S7). Under the 2100-BCC-CSM2-MR SSP585 scenario, the area of potential habitats of four *Juniperus* species (*J. formosana*, *J. pingii*, *J. squamata*, *J. coxii*, *J. indica*) would have a large loss (18.1–41.9%) compared to the present, while the area losses of *J. pingii* var. *wilsonii* and *J. saltuaria* would be smaller, 4.5% and 13.7%, respectively (Table S7). In addition, *J. formosana* would have the largest area loss with a high suitability of 0.6–1 and potential areas with a suitability of 0.2–1. The area overlapping with the present potential habitats would be high (49.9–71.4%), and a few non-overlapping areas would appear in the north (Figure 7E–H,L–N). Notably, the non-overlapping areas of *J. pingii* var. *wilsonii* and *J. saltuaria* would be larger than for the other five species.

Either under the 2100-BCC-CSM2-MR SSP126 scenario or under the BCC-CSM2-MR SSP585 scenario, the loss of potential suitable area of *J. squamata*, *J. formosana*, and *J. pingii* would show a larger loss than the other three species. Among them, the loss of area with high suitability of 0.6–1 of *J. formosana* was the greatest. Clearly, under the 2100-BCC-CSM2-MR SSP585 scenario, the percentage of loss of potential habitat would have a larger loss for *J. formosana* and *J. indica* compared to the 2100-BCC-CSM2-MR SSP126 scenario.

We considered the overlapping areas of potential habitats under the present climate and two climatic scenarios in the future as stable refugia for each of the seven *Juniperus* species as shown in Figure 7A–H and Figure 7I–N, respectively. The shared stable refugia of the *Juniperus* species would be mainly located in the Qinghai-Tibet Plateau and the Hengduan Mountains region.

## 4. Discussion

### 4.1. Habitats and Regeneration

The modern distribution pattern of *Juniper* communities in Yunnan shows that they are centered in northwestern Yunnan of the Hengduan Mountains region and some communities are scattered in central and western Yunnan, as shown in Figure 3. This distribution pattern reflects that the Hengduan Mountains region is the primary center of the modern distribution and diversification for *Juniperus* species in China [6,7]. The distribution pattern is closely related to their evolutionary history, which has been significantly influenced by ancient climate changes and specific geological events. Mao et al. [11] estimated the divergence times among *Juniperus* species, suggesting that the early divergent lineages of *Juniperus* were distributed in warmer habitats. *J. formosana*, which is an early divergent lineage, is found in evergreen coniferous forests at elevations of 2070–2208 m of Yunnan, typically near roads and villages on lower mountain tops, where environmental temperatures are relatively warm. In contrast, the lineages that diverged by the uplift of the Tibetan Plateau after the Miocene [6,70,71,72], including *J. pingii*, *J. coxii*, *J. squamata*, *J. saltuaria*, *J. pingii* var. *wilsonii*, and *J. indica*, are found in evergreen coniferous forests at elevations above 3000 m, where the environmental temperatures are lower [11]. High-altitude mountain habitats are characterized by lower temperatures and reduced soil moisture, which restrict the survival of non-cold-resistant and non-drought-resistant plants. Consequently, the species diversity within the communities of *J. pingii*, *J. coxii*, *J. squamata*, *J. saltuaria*, *J. pingii* var. *wilsonii*, and *J. indica* is lower than that of the *J. formosana* community. The species composition within these communities predominantly includes cold-resistant and drought-resistant alpine plants such as species from *Juniperus*, *Abies*, *Picea*, *Larix*, *Pinus*, *Quercus*, *Rhododendron*, and *Berberis*.

### 4.2. Species Diversity

An increase in species diversity within a particular community promotes long-term stability because that increased biodiversity could promote species asynchrony, thereby enhancing the resilience and stability of ecosystems to environmental change [73]. The *Juniperus* communities within this region exhibit low species richness, pronounced dominance species, and an extremely uneven distribution compared to communities dominated by *Pseudotsuga forrestii* [49] or *Taxus wallichiana* var. *wallichiana* in Yunnan [48]. As a result, the resilience of *Juniperus* communities in the face of environmental fluctuations might be weaker compared to other evergreen coniferous species such as *P*. *forrestii* and *T. wallichiana* var. *wallichiana*. Among the seven community types, Type 2 has the highest species richness and diversity indices, which may be related to its habitat environment. The lower altitude areas have better hydrothermal conditions, which can provide more ecological niches, and therefore their diversity indices are higher. For instance, Ali et al. [74] analyzed the relationship between vegetation diversity patterns in the subtropical region during spring with topographic and soil variables, finding that the communities in plains lying at lower altitudes had higher diversity while the communities formed at high altitudes had lower diversity.

### 4.3. Regeneration

Combining the GLM analysis of juveniles and saplings (Figure S4), we found that as the height of juveniles and saplings increased, their numbers showed a trend of decline, indicating that there are limitations to the regeneration ability of the *Juniperus* species within Yunnan. And the *Juniperus* species under study are distributed in limestone habitats, steep slopes, mountain slopes along roadsides, gullies and riversides, and a number of juveniles and saplings are primarily found in micro-habitats like roadsides and forest gaps, suggesting that *Juniperus* regeneration is associated with intermediate levels of disturbance—a pattern also observed in other evergreen coniferous species, such as *P. forrestii* and *T. wallichiana* var. *wallichiana* [48,49]. However, almost no established *Juniperus* juveniles and saplings (90–130 cm in height) were found, indicating that *Juniperus* regeneration has been weak in recent years.

### 4.4. Phylogenetic Diversity

The current species composition within a community is the result of both evolutionary and ecological processes, and analyzing the phylogenetic relationships among species can reflect the historical processes involved in the formation of existing communities [27]. During community assembly, habitat filtering aggregates closely related species with similar traits, resulting in phylogenetic clustering, whereas competitive exclusion drives species with similar traits to diverge, leading to phylogenetic overdispersion. Our analysis of seven *Juniperus* community types indicates that the phylogenetic relationships of *Juniperus* communities in Yunnan, excluding *J. formosana* forest type, are more distant than expected by chance (NRI < 0, NTI < 0), demonstrating an overdispersion phylogenetic pattern [27]. This suggested that the formation of communities in the other six community types might have been primarily driven by ecological niche differentiation or competitive exclusion. In other words, due to similar ecological niches, the plants that managed to survive in high-altitude mountainous areas might compete with each other for limited resources [75]. As a result, only some of them won out and assembled the current community.

Compared with the other six *Juniperus* community types, the *J. formosana* communities showed a more complex phylogenetic structure (NRI < 0, NTI > 0). This might be related to the influence of external disturbances, which could directly affect community dynamics and lead to phylogenetic clustering [76]. For example, Verdú et al. [77] and Ojeda et al. [78] found that the plant communities around the Mediterranean with high frequencies of wildfires exhibited phylogenetic clustering; conversely, communities with low wildfire frequencies tended to show overdispersion phylogenetic relationships. Similar to that example, Dinnage [79] also found that the phylogenetics of plant communities in abandoned land following human interference was more clustered than plant communities without human interference. In our study, the habitats of *J. formosana* evergreen coniferous forests at relative low altitudes are close to villages, where human activities are more frequent, resulting in a greater disturbance intensity compared to the other six community types, so the phylogenetics of *J. formosana* communities were more clustered (expressed by NTI > 0).

### 4.5. Management and Conservation Recommendations

Considering climate change, the drastic changes in temperature and precipitation in future under the SSP585 scenarios will increase the habitat fragmentation of *Juniperus* species, as a result, the loss of potential habitat areas with SSP585 scenarios will be larger than that under SSP126 scenarios. With global warming, most animals and plants are expected to migrate to higher latitudes and higher altitudes [80], including *Juniperus* species. Under two future climate scenarios, the loss of potential habitats area is in the south, while the seven *Juniperus* species would spread their potential habitats area to the north under the SSP585 scenario. Among them, *J*. *pingii* var. *wilsonii* and *J*. *saltuaria* show the most significant northward migration.

**Figure 8:**
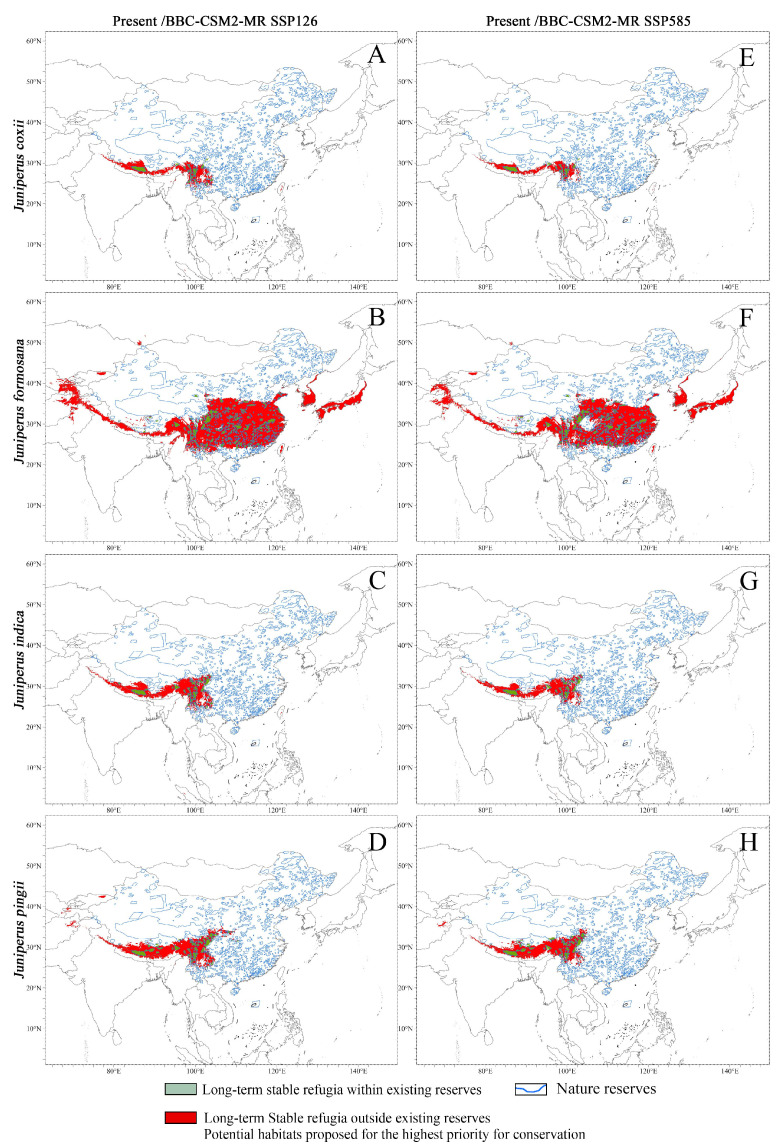

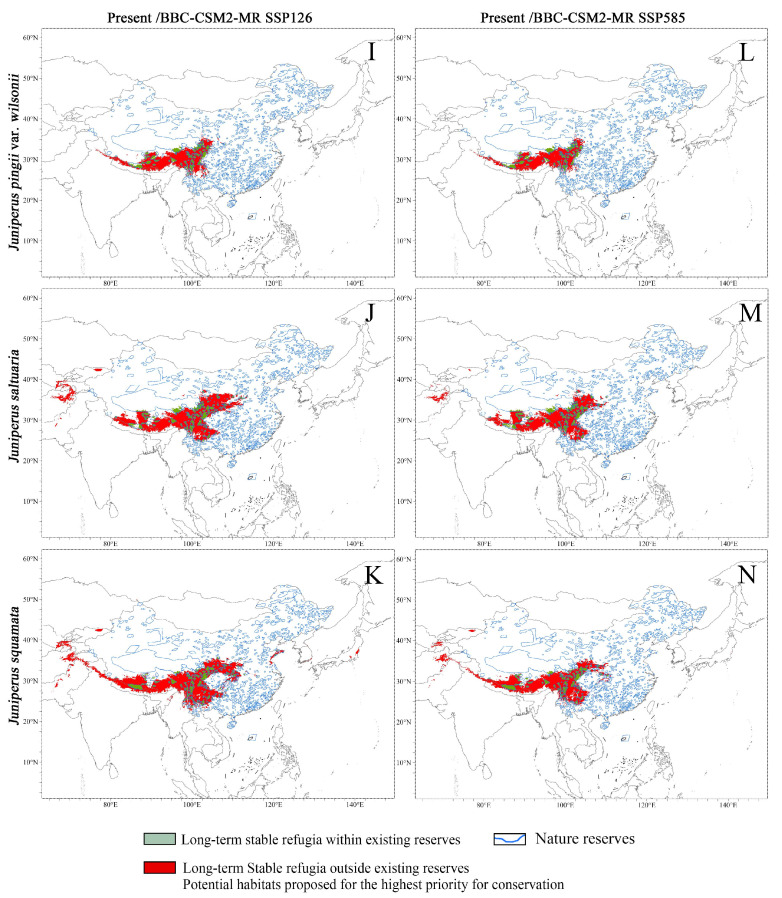
The overlap areas of potential habitats with and without protection in China under the present climate and two climatic scenarios in the future (2100). (**A**–**D**,**I**–**K**): Under scenarios of present–future 2100-BCC-CSM2-MR SSP126. (**E**–**H**,**L**–**N**): Under scenarios of present–future 2100-BCC-CSM2-MR SSP585.

Since the distribution range of *Juniperus* species is primarily within China, and also their future potential distribution is mainly in China, we only assessed priority protected areas in China in this study. We considered that the overlapping areas (the gray area in Figure 7) of the present and future are the climatic stable refugia of the seven *Juniperus* species. Our analysis of the stable refugia and nature reserves in China showed that only 13.6–35.1% stable refugia fell within protected areas (nature reserves) in China (Table 1 and Figure 8A–N), and 80% of stable refugia are not included within the protected areas. With global warming, this lack of in situ conservation will continue and may even worsen in the future. On average, 78.51% of the stable refugia in China would be outside the network of nature reserves in the two predicted models for the year 2100.

The most urgent needs for new nature reserves are for the shared stable refugia of the seven species, i.e., the Qinghai-Tibet Plateau and Hengduan Mountains region, including southwestern Sichuan, northwestern Yunnan, and southeastern and southern Xizang (southern Tibet), which are the main distribution areas of *Juniper* species with high suitability (Figure S5, Figure S6 Part1, Figure S6 Part2 and Table 1). For *J. indica* and *J. squamata*, which are mainly distributed in special habitats, we especially recommend in situ conservation. Notably, these predictions are solely based on climate change, and there are other dangers, such as human activities and vegetation fires.

Our classification of 131 vegetation plots into seven distinct *Juniperus*-dominated community types provides a clear framework for conservation planning. Certain communities, such as *J. pingii* communities and *J. coxii* communities, harbor a high proportion of threatened or endemic species and occur in small, fragmented habitats, making them high-priority targets for protection. Others, such as *J. squamata* communities and *J. pingii* var. *wilsonii* communities, occupy relatively intact but climate-sensitive habitats, where proactive management could prevent future degradation. Linking community types to these conservation priorities ensures that protection and restoration measures are tailored to the ecological characteristics and vulnerabilities of each community. In addition, in *J. formosana* communities, which are frequently disturbed by human activities, juveniles and saplings are nearly absent. To ensure the genetic resources and survival of these species, it is essential to implement some conservation work in advance, such as census species lists, collection of germplasm resources, and building seed banks. Additionally, planting seedlings of *Juniperus* in suitable habitats under future climatic conditions could be a successful conservation strategy [81].

### 4.6. Potential Limitations

One limitation of this study is that phylogenetic relatedness in plant communities was assessed using only two commonly used metrics—net relatedness index (NRI) and nearest taxon index (NTI)—which may not capture the full complexity of evolutionary relationships. In addition, we did not incorporate species’ functional traits due to limited available data. Another limitation is that potential species habitats were predicted solely based on climatic variables. Other important factors, such as anthropogenic disturbances, habitat availability, and dispersal constraints, were not included in the ecological niche modeling. Despite these limitations, our study provides a valuable foundation for understanding the *Juniperus* community structure, diversity, and future distribution under climate change. It offers critical baseline information that can be built upon by future research integrating broader ecological, functional, and anthropogenic factors.

## 5. Conclusions

The *Juniperus* communities in Yunnan Province are mainly composed of temperate components, with low species diversity and distant phylogenetic relationships, indicating that niche differentiation or competitive exclusion may be the main driving force for community formation. The regeneration ability of *Juniperus* species is weak, and they mainly rely on natural disturbances in unstable micro-habitats for regeneration.

Climate change will lead to a reduction in the potential habitat areas of *Juniperus* species, especially a significant loss of high suitability areas for *J. formosana* and *J. squamata*. In the future, the potential distribution area of *Juniperus* species will migrate northward, especially under the SSP585 scenario.

The current network of nature reserves fails to effectively cover the stable refuge areas of *Juniperus* species. It is recommended to establish new nature reserves in the Qinghai-Tibet Plateau and the Handgun Mountains to protect the genetic resources and survival environment of these species.

## Figures and Tables

**Table 1:** Predicted areas of stable refugia of the seven *Juniperus* species within and outside the network of nature reserves in China. JC, *J. coxii*, JF, *J. formosana*, JI, *J. indica,* JP, *J. pingii*, JPW, *J. pingii* var. *wilsonii*, JSA, *J. saltuaria*, JSQ, *J. squamata*.

		Area of Stable Refugia (×10^4^ km^2^)	Area of Stable Refugia Within Nature Reserves (×10^4^ km^2^ and %)	Area of Stable Refugia Outside Nature Reserve (×10^4^ km^2^ and %)
JC	Present	65.16	12.76 (19.58)	52.4 (80.42)
	BCC-CSM2-MR 126	50.46	10.69 (21.19)	39.77 (78.81)
	BCC-CSM2-MR 585	41.82	9.48 (22.66)	32.34 (77.34)
JF	Present	431.79	58.7 (13.59)	373.09 (86.41)
	BCC-CSM2-MR 126	393.32	54.82 (13.94)	338.5 (86.06)
	BCC-CSM2-MR 585	316.92	50.73 (16.01)	266.19 (83.99)
JI	Present	86.54	17.42 (20.13)	69.12 (79.87)
	BCC-CSM2-MR 126	82.20	16.66 (20.27)	65.54 (79.73)
	BCC-CSM2-MR 585	70.91	15.63 (22.04)	55.28 (77.96)
JP	Present	127.73	26.95 (21.1)	100.78 (78.9)
	BCC-CSM2-MR 126	96.63	20.72 (21.44)	75.91 (78.56)
	BCC-CSM2-MR 585	83.84	20.44 (24.37)	63.4 (75.63)
JPW	Present	105.16	26.25 (24.96)	78.91 (75.04)
	BCC-CSM2-MR 126	90.48	23.81 (26.31)	66.68 (73.69)
	BCC-CSM2-MR 585	100.48	35.25 (35.08)	65.23 (64.92)
JSA	Present	184.81	35.73 (19.33)	149.08 (80.67)
	BCC-CSM2-MR 126	154.81	31.47 (20.33)	123.34 (79.67)
	BCC-CSM2-MR 585	159.52	48.33 (30.3)	111.19 (69.7)
JSQ	Present	239.64	40.35 (16.84)	199.29 (83.16)
	BCC-CSM2-MR 126	172.32	31.66 (18.37)	140.66 (81.63)
	BCC-CSM2-MR 585	139.26	32.61 (23.41)	106.65 (76.59)

## Data Availability

The original contributions presented in this study are included in the article/Supplementary Material. Further inquiries can be directed to the corresponding author.

## Supplementary Materials

The following supporting information can be downloaded at: https://www.mdpi.com/2223-7747/14/17/2754/s1, Figure S1: Fossils of *Juniperus* from the Paleogene, Neogene and Quaternary; Figure S2: The best number of clusters for *Juniperus* community types; Figure S3: Variation in the density of juveniles and saplings of *Juniperus* species across different height classes in various micro-habitats; Figure S4: The GLM analysis of juveniles and saplings of *Juniperus* species; Figure S5: The potential habitats under the present climate scenarios in the future (2100); Figure S6, Part1 and Part2: The potential habitats under the two climatic scenarios in the future (2100); Table S1: Environmental characteristics of plots in each forest type; Table S2: Floristic areal types of families and genera of seed plants in the forests; Table S3: Species composition of the arborous layer (height ≥ 5 m) in each forest type; Table S4: Species composition of the shrub layer (5 m > height ≥ 1.3 m) in each forest type; Table S5: Species composition of the herb layer (height < 1.3 m) in each forest type; Table S6: Performance of models and variable contributions for each *Juniperus* species; Table S7: Predicted potential distribution areas of the seven *Juniperus* species for the present and two scenarios under future (2100) climate, and the overlap areas between the present and future.
