# Supplementary material for: Community and Population Characteristics and Future Potential Habitats Under Climate Change of Juniperus Species in Yunnan, Southwestern China
Source: Plants (Basel). 2025 Sep 3;14(17):2754. doi: 10.3390/plants14172754 (PMC12430647; doi:10.3390/plants14172754)
Supplement: Supplementary file 1 [file plants-14-02754-s001.zip › plants-3799156-supplementary.pdf]

Community characteristics, phynogenetic diversity and population structure and future potential habitats under climate change of *Juniperus* species in Yunnan, southwestern China

You-Cai Shi[1], Qing Chen[1], Min-Rui Du[1], Shu-Li Xiao[1], Shuai-Feng Li[2], Xiao-Fan Wang[1], Qiao Li[1], Cindy Q. Tang[1*]

[1] College of Ecology and Environmental Science, Yunnan University, Building #3, Guozhong Dalou, Chenggong Campus, Dongwaihuan South Road, University Town, Chenggong New District, Kunming 650504, China;

[2] Institute of Highland Forest Science, Chinese Academy of Forestry, Kunming 650224, China;

[*] Correspondence: cindytang@ynu.edu.cn

This file includes: Supplementary Tables S1-S7 and Supplementary Figure S1-S6.

Table S1 Environmental characteristics of plots in each forest type. LS: Low slope; MS: Middle slope; US: Upper slope; TM: Top of Mountain.

Plot	City/Perfecture	County/City	North latitude	East longitude	Elevation (m)	Aspect (°)	Inclination (°)	Terrain
Community Type 1								
P72	Kunming	Dongchuan	26.24151	102.9465	3193	ES153	30	MS
P73	Kunming	Dongchuan	26.24151	102.9465	3193	ES153	30	MS
P74	Diqing	Weixi	27.42411	99.15389	3049.04	ES154	28	US
P75	Diqing	Weixi	27.42454	99.15288	3062.36	ES143	32	US
P76	Diqing	Weixi	27.42527	99.15458	3058.17	ES167	30	US
P77	Lijiang	Ninglang	27.7535	100.749	3495.7	ES168	45	US
P78	Lijiang	Ninglang	27.7535	100.749	3495.7	ES169	45	US
P79	Lijiang	Ninglang	27.7535	100.749	3495.7	ES170	45	US
Community Type 2								
P95	Kunming	Xundian	25.75932	103.3224	2070	WN332	45	MS
P96	Kunming	Xundian	25.75932	103.3224	2070	WN332	45	MS
P97	Kunming	Xundian	25.77629	103.4956	2208	S175	20	TM
P98	Kunming	Xundian	25.77629	103.4956	2208	S175	20	TM
P99	Kunming	Xundian	25.77629	103.4956	2208	S175	20	TM
Community Type 3								
P100	Dali	Yangbi	25.74558	100.0305	3391.1	WS216	10	MS
P101	Dali	Yangbi	25.74558	100.0305	3391.1	WS216	10	MS
P102	Dali	Yangbi	25.74558	100.0305	3391.1	WS216	10	MS
P103	Dali	Yunlong	25.74722	99.04711	3125.11	WS207	20	US
P104	Dali	Yunlong	25.74743	99.05855	3171.93	WS207	32	US
P105	Dali	Yunlong	25.74746	99.05875	3170.13	WS214	35	US
P106	Dali	Yunlong	25.74755	99.05907	3174.34	WS209	27	US
P107	Dali	Yunlong	25.74756	99.05907	3175.46	WS206	28	US
P108	Dali	Yunlong	25.74826	99.05432	3157.1	WS206	32	US
P109	Nujiang	Lushui	25.99468	98.70991	3014.45	S163	65	TM
P110	Nujiang	Lushui	25.99468	98.70991	3014.45	S163	65	TM
P111	Nujiang	Lushui	25.99468	98.70991	3014.45	S163	65	TM
P112	Dali	Yunlong	26.08323	99.21381	3468.63	ES132	35	TM

P113	Dali	Yunlong	26.0835	99.21322	3468.63	ES140	25	TM
P114	Dali	Yunlong	26.08499	99.21095	3450.77	ES132	27	TM
P115	Dali	Yunlong	26.085	99.21094	3444.46	ES132	30	TM
P116	Dali	Yunlong	26.20523	99.23025	3261.7	WS249	30	US
P117	Nujiang	Fugong	26.55594	98.96925	3351.19	E111	45	TM
P118	Nujiang	Fugong	26.55594	98.96925	3351.19	E111	45	TM
P119	Nujiang	Fugong	26.55594	98.96925	3351.19	E111	45	TM
P120	Diqing	Weixi	27.14564	99.23675	3359.68	WN258	20	TM
P121	Diqing	Weixi	27.14799	99.23982	3367.02	WN307	26	TM
P122	Diqing	Weixi	27.15109	99.23635	3362.99	WN253	20	TM
P123	Diqing	Weixi	27.4709	99.2027	3359.91	WS200	45	US
P124	Diqing	Weixi	27.4709	99.20103	3389.23	ES134	32	US
P125	Diqing	Weixi	27.471	99.20021	3372.43	ES156	32	US
P126	Diqing	Deqin	27.77259	99.15725	3538.78	WS223	30	US
P127	Diqing	Deqin	27.77277	99.1563	3561.66	ES169	32	US
P128	Diqing	Deqin	27.77366	99.15423	3575.42	ES172	30	US
P129	Diqing	Weixi	27.84628	99.07285	3442.46	WS183	18	US
P130	Diqing	Weixi	27.85593	99.09314	3481.48	WS198	18	US
P131	Diqing	Weixi	27.85599	99.09346	3475.57	WS198	18	US
Community Type 4								
P19	Lincang	Yongde	24.08373	99.62694	3146.34	ES151	40	TM
P20	Lincang	Yongde	24.08626	99.62586	3142.34	E86	30	TM
P21	Lincang	Yongde	24.08626	99.62586	3142.34	E86	30	TM
P22	Lincang	Yongde	24.08626	99.62586	3142.34	E86	30	TM
P23	Lincang	Yongde	24.08676	99.62615	3142.34	ES151	40	TM
P24	Dali	Yangbi	25.79632	100.0096	3409	ES137	20	LS
P25	Dali	Yangbi	25.79632	100.0096	3377	ES142	20	LS
P26	Dali	Yangbi	25.79632	100.0096	3377	ES142	20	LS
P27	Kunming	Luquan	26.09019	102.8402	3868	W268	45	US
P28	Kunming	Luquan	26.09019	102.8402	3855	W268	45	US
P29	Kunming	Luquan	26.09019	102.8402	3880	W268	45	US
P30	Kunming	Luquan	26.09026	102.8433	3916	WS195	25	US
P31	Kunming	Luquan	26.09026	102.8433	3916	WS195	25	US
P32	Kunming	Luquan	26.09026	102.8433	3916	WS195	25	US
P33	Kunming	Luquan	26.095	102.846	4050	W268	45	US
P34	Kunming	Luquan	26.095	102.846	4050	W268	45	US
P35	Kunming	Luquan	26.095	102.846	4050	W268	45	US
P36	Kunming	Luquan	26.1162	102.858	3612	W259	50	US
P37	Kunming	Luquan	26.1256	102.8582	3636	W268	45	US
P38	Kunming	Luquan	26.1532	102.8448	3686	WS202	45	TM
P39	Kunming	Luquan	26.1535	102.8461	3667	S3	45	TM
P40	Kunming	Luquan	26.1552	102.8471	3652	WS261	35	TM
P41	Diqing	Weixi	27.61037	99.31482	3619	ES161	28	US
P42	Diqing	Weixi	27.61045	99.31358	3628.92	WS216	36	US
P43	Diqing	Weixi	27.61537	99.31649	3623.72	ES219	28	US
P44	Lijiang	Ninglang	27.6424	100.8138	3196	WS192	60	MS
P45	Diqing	Weixi	27.82368	98.97432	3462.58	WS216	30	US
P46	Diqing	Weixi	27.96426	98.97631	3456.57	WS242	35	US

P47	Diqing	Weixi	27.97395	98.9562	3462.04	WS234	35	US
P48	Diqing	Weixi	27.98865	99.42535	3530.65	WS235	20	US
P49	Diqing	Weixi	27.99795	99.4278	3537.63	ES172	20	US
P50	Diqing	Weixi	27.99917	99.42679	3536.75	WS242	20	US
P51	Diqing	Deqin	28.60403	98.67546	3952	WS190	40	US
P52	Diqing	Deqin	28.60403	98.67546	3952	WS190	40	US
P53	Diqing	Deqin	28.60403	98.67546	3952	WS190	40	US
Community Type 5								
P54	Diqing	Deqin	28.32554	99.12213	4152	WS213	35	US
P55	Diqing	Deqin	28.32554	99.12213	4152	WS213	35	US
P56	Diqing	Deqin	28.32554	99.12213	4152	WS213	35	US
P57	Diqing	Deqin	28.33224	99.10243	4130.8	WS256	45	US
P58	Diqing	Deqin	28.33224	99.10243	4130.8	WS256	45	US
P59	Diqing	Deqin	28.33224	99.10243	4130.8	WS256	45	US
P60	Diqing	Deqin	28.33376	99.08866	4175	ES171	45	US
P61	Diqing	Deqin	28.33462	99.08837	4198	ES171	45	US
P62	Diqing	Deqin	28.33482	99.08822	4172	ES171	45	US
P63	Diqing	Deqin	28.37146	99.02433	4225.7	S183	38	US
P64	Diqing	Deqin	28.37215	99.02522	4210.9	S183	43	US
P65	Diqing	Deqin	28.37526	99.02473	4230.78	S165	32	US
P66	Diqing	Deqin	28.61808	98.72743	2791	EN25	50	US
P67	Diqing	Deqin	28.61808	98.72743	2791	EN26	50	US
P68	Diqing	Deqin	28.61808	98.72743	2791	EN35	50	US
P69	Diqing	Deqin	28.91105	98.93554	4225	WS229	35	MS
P70	Diqing	Deqin	28.91105	98.93554	4225	WS230	35	MS
P71	Diqing	Deqin	28.91105	98.93554	4225	WS231	35	MS
Community Type 6								
P1	Dali	Yangbi	25.75242	100.0422	3854.8	WS212	25	TM
P2	Dali	Yangbi	25.75242	100.0422	3854.8	WS212	25	TM
P3	Dali	Yangbi	25.75242	100.0422	3854.8	WS212	25	TM
P4	Nujiang	Lushui	26.4367	98.74463	3651.3	ES135	45	TM
P5	Nujiang	Lushui	26.4367	98.74463	3651.3	ES135	45	TM
P6	Nujiang	Lushui	26.4367	98.74463	3651.3	ES135	45	TM
P7	Diqing	Weixi	27.62074	99.11073	3853.57	ES146	30	US
P8	Diqing	Weixi	27.62186	99.11234	3864.64	ES139	25	US
P9	Diqing	Weixi	27.62325	99.11238	3872.58	ES146	30	US
P10	Diqing	Weixi	27.63453	99.13474	3958.45	ES131	25	US
P11	Diqing	Weixi	27.63862	99.13685	3961.32	ES131	25	US
P12	Diqing	Weixi	27.63881	99.13768	3958.27	ES122	18	US
P13	Diqing	Deqin	28.32865	99.12595	4221.7	WS194	50	US
P14	Diqing	Deqin	28.32865	99.12595	4221.7	WS194	50	US
P15	Diqing	Deqin	28.32865	99.12595	4221.7	WS194	50	US
P16	Diqing	Deqin	28.35881	99.06597	4239	WS240	15	MS
P17	Diqing	Deqin	28.35881	99.06597	4239	WS240	15	MS
P18	Diqing	Deqin	28.35881	99.06597	4239	WS240	15	MS
Community Type 7								
P80	Diqing	Xianggelila	28.1307	99.83462	4015.5	ES192	60	US

P81	Diqing	Xianggelila	28.1307	99.83462	4015.5	ES192	60	US
P82	Diqing	Xianggelila	28.1307	99.83462	4015.5	ES192	60	US
P83	Diqing	Xianggelila	28.13073	99.87229	3968.33	WS208	32	US
P84	Diqing	Xianggelila	28.13123	99.84789	4020.36	ES192	56	US
P85	Diqing	Xianggelila	28.13123	99.84789	4020.36	ES192	56	US
P86	Diqing	Deqin	28.33181	99.0195	3987.1	S180	25	LS
P87	Diqing	Deqin	28.33181	99.0195	3987.1	S180	25	LS
P88	Diqing	Deqin	28.33181	99.0195	3987.1	S180	25	LS
P89	Diqing	Deqin	28.33398	99.03859	3900.5	WS188	20	LS
P90	Diqing	Deqin	28.33399	99.04125	3910.3	WS169	20	LS
P91	Diqing	Deqin	28.33402	99.0395	3945.4	WS210	30	LS
P92	Diqing	Deqin	28.33894	99.0421	3929.7	WS202	30	LS
P93	Diqing	Deqin	28.34184	99.03059	4046.5	S176	25	LS
P94	Diqing	Deqin	28.34219	99.02991	3998.7	ES164	20	LS

Table S2 Floristic areal types of families and genera of seed plants in the forests

	The areal types	Number of families	Proportion (%)	Number of genera	Proportion (%)
1	Widespread	(27)		(26)	
2	Pantropic	15	34.1	27	14.1
2-1	Trop. Asia - Australasia and Trop. Amer. (S. Amer. or / and Mexico)				
2-2	Trop. Asia - Trop. Afr. - Trop. Amer. (S. Amer.)	1	2.3		
2S	Pantropic especially S. Hemisphere	1	2.3		
3	Trop. & Subtr. E. Asia & (S.) Trop. Amer. disjuncted	3	6.8	2	1.1
3b	Trop. & Subtr. C. to S. Amer., including C. Mexico &W.I.				
4	Old World Tropics = OW Trop.	1	2.3	3	1.6
5	Trop. Asia to Trop. Australasia Oceania	1	2.3	1	0.5
6	Trop. Asia to Trop. Africa			7	3.7
6-2	Trop. Asia & E. Afr. Or Madagasca disjuncted				
6d	S. Afr., chiefly Cape	1	2.3		
7	Trop. Asia			3	1.6
7-1	Java or Sumatra, Himalaya to S., SW. China disjuncted or diffused			2	1.1
7-3	Myanmar, Thailand to SW. China				
7-4	Vietnam or Indochinese Peninsula to S. or SW. China				
7a	W. Malesia beyond New Wallace line				
7b	C. Malesia				
7d	New Geainea				
8	N. Temp.	6	13.6	65	34.0
8-1	Circumpolar (Circumarctic).			1	0.5
8-2	Arctic - Alpine			4	2.1
8-4	N. Temp. & S. Temp. disjuncted	10	22.7	17	8.9
8-5	Eurasia & Temp. S. Amer. disjuncted	2	4.6	1	0.5
8-6	Mediterranea, E. Asia, N.Z. and Mexico-Chile disjuncted	1	2.3	1	0.5
9	E. Asia & N. Amer. disjuncted			10	5.2
10	Old World Temp. = Temp. Eurasia			13	6.8

10-1	Mediterranea, W. Asia (or C. Asia) & E. Asia disjuncted			3	1.6
10-3	Eurasia & S. Africa (Sometimes also Australasia) disjuncted.			1	0.5
11	Temp. Asia			2	1.1
12	Medit., W. to C. Asia			2	1.1
12-1	Mediterranea to C. Asia and S. Afr. And/or Australasia disjuncted				
12-3	Mediterranea to Temp. - Trop. Asia, with Australasia and/or S.N. to S. Amer. disjuncted			1	0.5
12-4	Mediterranea to Trop. Africa & Himalaya disjuncted.			1	0.5
13-2	C. Asia to Himalaya & S. W. China.			2	1.1
14	E. Asia			6	3.1
14SH	Sino - Himalaya	1	2.3	14	7.3
14SJ	Sino - Japan	1	2.3	2	1.1
15	Endemic to China			4	2.1
	Total	71(44)	100	217(191)	100

Table S3 Species composition of the arborous layer (height ≥ 5 m) in each forest type. Species with a relative importance value (RIV) ≥ 0.01% are shown.

Community type	Type 1	Type 2	Type 3	Type 4	Type 5	Type 6	Type 7
Number of species	7	4	25	21	3	1	6
Species	RIV (%)	RIV (%)	RIV (%)	RIV (%)	RIV (%)	RIV (%)	RIV (%)
Juniperus pingii	75.47			0.08			
Quercus senescens	10.59						
Pinus armandii	4.84	14.35	2.73	4.16			
Quercus longispica	4.59						
Elsholtzia fruticosa	2.21						
Picea brachytyla var. *complanata*	2.03		9.65	10.04			
Acer caesium	0.26						
Juniperus formosana		65.17					
Pinus yunnanensis		19.02	0.06	0.72			
Quercus franchetii		1.46					
Juniperus coxii			56.18	5.85			
Abies georgei			9.49	8.45	2.30	100.00	4.45
Tsuga dumosa			3.92				
Rhododendron aganniphum			3.65				
Rhododendron yunnanense			3.22	0.75			
Rhododendron irroratum			3.00				
Acer caudatum			1.25				
Rhododendron rubiginosum			1.01				1.55
Abies delavayi			0.75				
Rhododendron alutaceum			0.64				
Rhododendron decorum			0.63				
Heptapleurum heptaphyllum			0.62				
Rhododendron gongshanense			0.60				
Prunus tomentosa			0.47	0.59			
Yushania pianmaensis			0.44				

Abies nukiangensis			0.39				
Cerasus tomentosa			0.36				
Viburnum congestum			0.31				
Juniperus squamata			0.28	57.66			
Betula delavayi			0.24				
Acer palmatum			0.07				
Betula alnoides			0.06	0.17			
Rhododendron sphaeroblastum var. *wumengense*				2.10			
Rhododendron wardii				2.09			
Quercus pannosa				1.98			7.06
Zanthoxylum bungeanum				1.93			
Salix balfouriana				1.25			
Lonicera webbiana				0.79			
Rhododendron taliense				0.47			
Rhododendron cyanocarpum				0.37			
Hydrangea aspera				0.37			
Abies forrestii				0.10			
Abies georgei var. *smithii*				0.07			5.26
Juniperus saltuaria					85.70		
Larix potaninii var. *macrocarpa*					12.01		8.96
Juniperus indica							72.73

Table S4 Species composition of the shrub layer (5 m > height ≥ 1.3 m) in each forest type. Species with a relative importance value (RIV) ≥ 0.01% are shown.

Community type	Type 1	Type 2	Type 3	Type 4	Type 5	Type 6	Type 7
Number of species	21	18	41	68	34	20	22
Species	RIV (%)	RIV (%)	RIV (%)	RIV (%)	RIV (%)	RIV (%)	RIV (%)
Hypericum forrestii	30.29						
Juniperus pingii	26.72			0.23			
Elsholtzia fruticosa	8.36						
Berberis franchetiana	5.50		2.81	1.16	12.36		8.79
Cotoneaster hebephyllus	5.02				0.24		
Zanthoxylum bungeanum	3.85			0.53			
Hypericum monogynum	3.08						
Juniperus coxii	2.49		45.10	3.36			
Rosa soulieana	2.22			0.11			
Pinus armandii	1.93	1.12	2.97	0.89			
Hypericum acmosepalum	1.72	0.96		1.16			
Cirsium chlorolepis	1.65						
Cotoneaster buxifolius	1.39	7.52					
Berberis wilsonae	1.34			1.86	8.39	1.61	8.00
Juniperus squamata	1.20		0.27	54.69	1.51	2.28	
Buddleja asiatica	0.84						
Rubus niveus	0.83						
Quercus senescens	0.57						
Buddleja officinalis	0.50	1.80					

Cotoneaster franchetii	0.26		0.37	0.16	2.33		
Picea brachytyla var. *complanata*	0.24		3.29	3.17			0.94
Juniperus formosana		26.06					
Viburnum congestum		14.54	0.45				
Pyracantha fortuneana		13.02					
Berberis pruinosa		10.70					
Coriaria nepalensis		4.19					
Quercus acutissima		3.92					
Myrsine africana		3.72					
Pinus yunnanensis		3.58		0.16			
Keteleeria evelyniana		2.10					
Rhamnus aurea		2.00					
Spiraea martini		1.69					
Campylotropis polyantha		1.49					
Elsholtzia rugulosa		1.35					
Pistacia weinmanniifolia		0.24	0.14				
Yushania pianmaensis			9.43			15.25	
Rhododendron irroratum			5.62	1.20			
Rhododendron aganniphum			3.15				
Rhododendron decorum			3.06	0.15			
Yushania levigata			3.03				
Rhododendron alutaceum			2.79				
Rhododendron yunnanense			1.95	0.74			
Abies georgei			1.87	0.74	0.31	0.46	6.31
Fargesia frigidis			1.79				
Rhododendron rubiginosum			1.75	0.99		0.08	7.98
Ilex perryana			1.40				
Fargesia communis			1.30	7.52			
Rhododendron gongshanense			1.17				
Piptanthus nepalensis			1.02	0.31			
Rosa sweginzowii			0.61	0.92			5.99
Spiraea japonica			0.58	0.34			
Hypericum elodeoides			0.57				
Berberis insignis subsp. *incrassata*			0.52				
Rhododendron nivale subsp. *australe*			0.45				
Rhododendron sinogrande			0.37				
Cotoneaster microphyllus			0.37	0.06			
Daphne papyracea			0.37	0.13			
Gaultheria fragrantissima			0.32	0.25			
Abies nukiangensis			0.28				
Yushania niitakayamensis			0.22				
Acer caudatum			0.14				
Dryopteris sublacera			0.10				
Berberis ferdinandi-coburgii			0.10				
Tsuga dumosa			0.08				
Rubus niveus			0.07				

Yushania polytricha	0.06				
Hydrangea davidii	0.04				
Prunus tomentosa	0.04	0.03			
Berberis dictyophylla		2.30			
Rhododendron taliense		1.59		2.63	
Quercus pannosa		1.35			5.27
Rhododendron sikangense var. *exquisitum*		1.30			
Rhododendron sphaeroblastum var. *wumengense*		1.09			
Rhododendron wardii		0.86			
Spiraea schneideriana		0.84	2.56		4.30
Rosa soulieana var. *yunnanensis*		0.79	0.63		
Cotoneaster rubens		0.79	0.50		
Ribes glaciale		0.71	2.61		2.37
Quercus spinosa		0.60			
Rhododendron cyanocarpum		0.56			
Rhododendron martinianum		0.55			
Paeonia suffruticosa		0.53			
Deutzia monbeigii		0.45			
Lonicera tangutica		0.43			0.35
Quercus longispica		0.43			
Salix balfouriana		0.42	0.12		3.29
Lonicera webbiana		0.40			
Ribes alpestre var. *giganteum*		0.36			
Lithocarpus variolosus		0.30			
Viburnum cylindricum		0.28			
Osmanthus suavis		0.25			
Lonicera nigra		0.25			
Dasiphora fruticosa		0.22	2.85	2.58	1.25
Fargesia melanostachys		0.22			
Berberis amabilis		0.21			
Rhododendron flavidum		0.13	3.14	11.41	
Prinsepia utilis		0.13			
Buddleja lindleyana		0.12			
Hydrangea aspera		0.11			
Salix tenella		0.07			
Viburnum betulifolium		0.07	0.85		
Excoecaria acerifolia		0.07			
Rubus subornatus		0.06			
Ribes himalense		0.06			
Cercis chinensis		0.04			
Abies forrestii		0.04			
Clematis florida		0.04			
Saussurea parviflora		0.04			
Lonicera japonica		0.03			
Betula alnoides		0.03			
Cotoneaster adpressus		0.02			

Juniperus saltuaria					20.50	0.07	
Juniperus pingii var. *wilsonii*					17.10	48.64	2.36
Ostryopsis nobilis					8.33		
Rhododendron phaeochrysum					4.65	0.50	12.99
Rhododendron rupicola var. *chryseum*					3.02	8.62	0.74
Cotoneaster multiflorus					2.69		
Dasiphora arbuscula					2.40		
Deutzia hookeriana					0.83		
Larix potaninii var. *macrocarpa*					0.52	0.33	1.08
Caragana jubata					0.37		
Sageretia horrida					0.24		
Indigofera tinctoria					0.20		
Sophora davidii var. *chuansiensis*					0.15		
Cercidiphyllum japonicum					0.15		
Abelia uniflora					0.10		
Jasminum nudiflorum					0.09		
Daphne longilobata					0.08		
Salix balfouriana					0.07		
Quercus semecarpifolia					0.05		
Clematis montana					0.04		0.12
Dasiphora glabra						1.53	
Ribes tenue						1.10	
Ribes longiracemosum						0.96	
Juniperus indica						0.79	17.09
Ribs longeracemosum var. *longeracemosum*						0.70	
Rosa omeiensis						0.24	
Rhododendron lapponicum						0.23	
Lonicera trichosantha							9.19
Abies georgei var. *smithii*							0.91
Lespedeza forrestii							0.56
Salix daliensis							0.12

Table S5 Species composition of the herb layer (height < 1.3 m) in each forest type. Species with a relative importance value (RIV) ⩾ 0.01% are shown.

Community type	Type 1	Type 2	Type 3	Type 4	Type 5	Type 6	Type 7
Number of species	101	55	122	139	91	74	73
Species	RIV (%)	RIV (%)	RIV (%)	RIV (%)	RIV (%)	RIV (%)	RIV (%)
Fragaria vesca	10.28		0.05	4.73	5.23		7.85
Hydrocotyle nepalensis	5.43						
Potentilla chinensis	4.54		4.20	2.74	1.91	5.47	2.41
Hypericum forrestii	3.79						
Pilea sinofasciata	3.75						
Erysimum amurense	2.94						
Geranium nepalense	2.86						
Corydalis taliensis	2.47			0.14			

Galium odoratum	2.46						
Athyrium biserrulatum	2.40		3.18	1.71			
Trifolium repens	2.22	2.43		1.62			
Cotoneaster franchetii	2.18		1.01	1.34	2.29		2.49
Tussilago farfara	2.16						
Thalictrum cirrhosum	2.08						
Pteris dactylina	2.06			0.13			
Impatiens arguta	1.94						
Galium asperifolium	1.92	0.35			0.25		
Salvia japonica	1.91						
Persicaria nepalensis	1.85		4.72				
Clinopodium chinense	1.84		0.14	0.23			0.15
Deyeuxia scabrescens	1.78	0.35					
Juniperus pingii	1.60						
Juncus effusus	1.36		0.84	0.15		4.09	
Arenaria orbiculata	1.33						
Valeriana jatamansi	1.32						
Corydalis melanochlora	1.26						
Dactylis glomerata	1.09						
Anemone rivularis	1.03	0.87					
Ainsliaea henryi	1.01		0.95				0.22
Galinsoga parviflora	0.97						
Prunella vulgaris	0.96		0.16	0.21			
Berberis franchetiana	0.92		0.47	0.29	0.78		0.75
Arisaema erubescens	0.84	0.16					
Bistorta vivipara	0.83		4.00	4.56	3.18	4.49	3.88
Rubia cordifolia	0.73		0.06	0.18			
Artemisia princeps	0.73	2.91					
Artemisia lavandulifolia	0.71			0.51	1.89		
Avena fatua	0.70						
Clinopodium megalanthum	0.68	1.58					
Artemisia selengensis	0.67						
Viola biflora	0.67		2.62	2.92		6.32	
Cynoglossum lanceolatum	0.67						
Galium spurium	0.66						
Onychium japonicum var. *lucidum*	0.64		0.09	0.31			
Reineckea carnea	0.64						
Rumex nepalensis	0.55		1.12				
Saxifraga filicaulis	0.54						
Taraxacum mongolicum	0.54						
Bistorta sinomontana	0.53			0.10			
Argentina lineata	0.53	0.09		1.69	1.25		
Cirsium chlorolepis	0.51						
Fagopyrum gracilipes	0.51						
Ephedra saxatilis	0.50						
Galium hoffmeisteri	0.48			0.62			

Clinopodium polycephalum	0.43						
Thalictrum delavayi	0.43						
Gentiana otophora	0.42		0.08	0.31			
Pinus armandii	0.41		0.24	0.27			
Ligularia rumicifolia	0.40				2.28	1.63	4.09
Trachyspermum scaberulum	0.39						
Hypericum acmosepalum	0.38	0.11		0.35			
Plantago asiatica	0.37		1.13	0.05			
Primula pinnatifida	0.35			0.02			
Dumasia yunnanensis	0.34						
Potentilla hypargyrea	0.33				3.84	1.24	0.04
Anaphalis nepalensis	0.33		0.75	0.65	1.11	2.94	2.11
Geranium hispidissimum	0.32						
Pedicularis labordei	0.32						
Torilis japonica	0.31	0.09					
Aletris spicata	0.31						
Geranium carolinianum	0.31						
Ligularia dentata	0.28						
Silene viscidula	0.27	0.85					
Artemisia japonica	0.25						
Geranium wilfordii	0.24		1.25	0.68	1.79	0.72	3.21
Arabis paniculata	0.23			0.61			
Juncus allioides	0.22				1.83	2.29	3.45
Juncus concinnus	0.22						
Thalictrum aquilegiifolium var. *sibiricum*	0.20		0.27	0.35			0.27
Juniperus coxii	0.19		2.06	0.31			
Cotoneaster buxifolius	0.19	0.63					
Urtica mairei	0.18						
Myriactis nepalensis	0.16						
Ainsliaea bonatii	0.16	0.98					
Aster oreophilus	0.13		0.11				
Halenia elliptica	0.12		0.29	1.64	2.68		0.56
Geranium delavayi	0.12						
Stellaria aquatica	0.12						
Epilobium hirsutum	0.12			0.30	0.56	3.41	0.06
Rubia podantha	0.11	0.28					
Picea brachytyla var. *complanata*	0.11		0.78	0.31			
Sambucus javanica	0.11			0.30			
Silene gracilicaulis	0.11						
Gentiana macrophylla	0.09			0.31			
Arisaema franchetianum	0.09			2.60			
Heracleum candicans	0.09						
Dipsacus asper	0.05			0.73			
Koenigia campanulata	0.05						
Picris hieracioides	0.05						
Taraxacum officinale	0.05						

Carex nubigena	0.03						
Heteropogon contortus		13.98					
Origanum vulgare		7.78		0.07			
Lespedeza cuneata		6.82					
Cyperus cyperoides		6.40	1.53				
Capillipedium assimile		5.86					
Imperata cylindrica		5.34					
Leontopodium sinense		5.25					0.36
Elsholtzia rugulosa		5.22					
Incarvillea arguta		4.20					
Themeda triandra		3.98					
Cynodon dactylon		2.82					
Bidens pilosa		2.61					
Agrimonia pilosa		2.07					
Barleria cristata		1.44					
Rubus niveus		1.35					
Artemisia stechmanniana		1.20			3.30		
Arundinella setosa		1.20					
Saccharum rufipilum		1.16					
Verbena officinalis		1.11					
Leontopodium andersonii		1.10				0.50	
Chamaecrista leschenaultiana		1.07					
Stellaria vestita		1.01					
Leontopodium dedekensii		0.93					
Boenninghausenia albiflora		0.58					
Stellaria yunnanensis		0.41					
Justicia procumbens		0.40					
Myrsine africana		0.37					
Clematis peterae		0.36					
Arisaema yunnanense		0.32					
Galium elegans		0.32					
Oxalis corniculata		0.25					
Pteridium revolutum		0.25					
Coriaria nepalensis		0.20					
Setaria viridis		0.17					
Clematis chrysocoma		0.16					
Dioscorea hemsleyi		0.16					
Rubia oncotricha		0.15	0.26	0.26			
Spiraea martini		0.11					
Berberis julianae		0.09					
Erigeron sumatrensis		0.09					
Vicia sativa		0.07					
Rubus fockeanus			14.76	3.86		2.41	
Roscoea cautleoides			6.00				
Argentina lineata			4.70				
Fragaria nilgerrensis			4.02	4.12			

Carex baccans	3.96		2.21		
Iris bulleyana	2.59	1.88	2.26		0.40
Swertia macrosperma	2.28				
Poa annua	1.96	3.29			0.17
Cnidium monnieri	1.76	0.32			
Gentiana panthaica	1.31				
Hemiphragma heterophyllum	1.13	1.68	4.26		1.21
Veronica polita	1.01				
Lysimachia deltoidea var. *cinerascens*	0.96				
Lycopodiastrum casuarinoides	0.93				
Fragaria moupinensis	0.85				
Viola striatella	0.83				
Anaphalis aureopunctata	0.83	0.10	1.32	4.61	
Dryopteris sublacera	0.82	0.06			
Roscoea tibetica	0.79				
Carpesium abrotanoides	0.74				
Dichrocephala integrifolia	0.73				
Cotoneaster microphyllus	0.70				
Gymnadenia conopsea	0.62				
Arthraxon hispidus	0.58				
Hypericum elodeoides	0.58				
Carum carvi	0.55				
Lycopodium japonicum	0.55				
Arisaema elephas	0.50			0.99	
Rhododendron yunnanense	0.48	0.12			
Elatostema obtusum	0.43				
Abies georgei	0.43	0.24	0.05	0.03	1.16
Maianthemum japonicum	0.43				
Halenia corniculata	0.43			0.07	
Rhododendron irroratum	0.39				
Pedicularis rex	0.38		0.38		
Ligularia sibirica	0.38				
Smilax china	0.37	0.33			
Ilex perryana	0.36				
Anaphalis sinica	0.35				
Persicaria capitata	0.34	0.04			
Stellaria media	0.33	0.23			
Ligularia hookeri	0.27			2.01	
Persicaria chinensis	0.27				
Gentiana cephalantha	0.26				
Gaultheria fragrantissima	0.24	0.11			
Fargesia spathacea	0.24				
Ribes tenue var. *tenue*	0.22				
Persicaria microcephala	0.22				
Goodyera schlechtendaliana	0.20	0.10			
Berberis insignis subsp. *incrassata*	0.20				

Yushania pianmaensis	0.19				
Nasturtium officinale	0.19				
Fargesia frigidis	0.19				
Smilax scobinicaulis	0.18				
Rubus yunnanicus	0.16				
Yushania levigata	0.15				
Clinopodium gracile	0.15				
Rhododendron aganniphum	0.14				
Odontostemma pogonanthum	0.14				
Rhododendron decorum	0.13				
Rhododendron alutaceum	0.12				
Dryopteris wallichiana	0.12	0.15			
Mahonia napaulensis	0.10				
Yushania niitakayamensis	0.10				
Aconitum taronense	0.09				
Artemisia neosinensis	0.09				
Clematis montana	0.08				
Sibbaldianthe bifurca	0.08				
Polygonatum cirrhifolium	0.08		0.26	0.11	0.52
Maianthemum henryi	0.07				
Cardamine occulta	0.07				
Lilium lancifolium	0.07				
Persicaria glacialis	0.05	0.10			0.85
Panax notoginseng	0.05				
Pteridium aquilinum var. *latiusculum*	0.05				
Satyrium nepalense	0.05				
Juniperus squamata	0.04	3.30	0.34	0.40	
Anaphalis margaritacea	0.04				
Persicaria maculosa	0.04				
Paris polyphylla var. *yunnanensis*	0.04				
Galium hoffmeisteri	0.03				
Eleutherococcus leucorrhizus	0.03				
Chimaphila japonica	0.03	0.22			
Abies nukiangensis	0.02				
Rhododendron gongshanense	0.02				
Spiraea salicifolia	0.02	0.11			
Rumex acetosa	0.02				
Acer palmatum	0.02				
Pedicularis densispica	0.02				
Potentilla supina	0.02				
Ainsliaea latifolia	0.02				
Parnassia palustris	0.01				
Acer caudatum	0.01				
Trigonotis peduncularis	0.01				
Berberis wilsonae	0.01	0.07	1.45	0.29	0.73
Koenigia campanulata var. *fulvida*		8.36			

Epilobium amurense	3.33			
Argentina phanerophlebia	3.23			
Cotoneaster adpressus	2.50			
Geranium strictipes	2.19			
Adiantum capillus-veneris	2.19			
Juncus leucanthus	1.68			
Dolomiaea souliei var. *cinerea*	1.54	2.44	0.04	
Gentiana nanobella	1.47	0.72	0.57	
Luzula plumosa	1.32			
Bistorta macrophylla	1.27	0.19		
Rhodiola yunnanensis	1.06			
Saussurea japonica	0.97	0.24		
Swertia cincta	0.95	0.29		0.02
Roscoea alpina	0.87			
Saussurea parviflora	0.78			
Ponerorchis chusua	0.78	1.04	3.52	
Buddleja microstachya	0.72			
Circaea alpina	0.67			3.69
Youngia japonica	0.58			
Gentiana scabra	0.51		3.41	
Ribes glaciale	0.49			0.04
Bassecoia hookeri	0.44	0.47		
Hymenidium foetens	0.44	1.08	0.05	
Selaginella tamariscina	0.37			
Quercus pannosa	0.33			0.13
Aconitum brevicalcaratum	0.30	1.84		1.80
Rosa omeiensis	0.30			
Primula polyneura	0.29	11.07	3.72	11.57
Salvia flava	0.28			0.98
Sedum stellariifolium	0.27			
Impatiens balsamina	0.24		0.51	
Salvia przewalskii	0.24			
Pedicularis macrosiphon	0.22			
Berberis lijiangensis	0.21			
Saxifraga stolonifera	0.21			
Deutzia monbeigii	0.21			
Galium bungei	0.21			
Paeonia 脳 *suffruticosa*	0.20			
Synotis cappa	0.16			
Fargesia melanostachys	0.16			
Pilea notata	0.16			
Salix balfouriana	0.15			
Primula sinolisteri	0.14			
Liparis campylostalix	0.14			
Rumex japonicus	0.13			
Berberis dictyophylla	0.12			

Pinus yunnanensis	0.11			
Polygonatum verticillatum	0.10			
Hypericum monogynum	0.10			
Ribes himalense	0.09			
Cyclosorus dentatus	0.09			
Draba yunnanensis	0.09	0.20		
Rhododendron wardii	0.08			
Oxyria digyna	0.08		1.96	
Saxifraga strigosa	0.08			
Geranium sibiricum	0.08			
Quercus spinosa	0.08			
Anemone flaccida	0.07			
Rhamnus davurica	0.06			
Viburnum betulifolium	0.06			
Geranium himalayense	0.05		3.96	
Artemisia caruifolia	0.05			
Pinellia ternata	0.05			
Zanthoxylum bungeanum	0.05			
Balanophora fungosa	0.05			
Rhododendron sikangense var. *exquisitum*	0.04			
Balanophora involucrata	0.04			
Pseudognaphalium adnatum	0.04			
Epipactis helleborine	0.04			
Silene napuligera	0.04			
Meconopsis speciosa	0.04			
Berberis amabilis	0.04			
Heptapleurum heptaphyllum	0.03			
Hypopitys monotropa	0.03			
Oenanthe javanica	0.03			
Cercis chinensis	0.03			
Sinopodophyllum hexandrum	0.02	0.26		3.11
Polygonatum sibiricum	0.01	0.50		0.61
Dryopteris sinofibrillosa	0.01			
Juniperus pingii var. *wilsonii*		4.08	3.89	3.96
Primula sikkimensis		3.11	1.38	2.84
Caltha sinogracilis		2.90		2.99
Davallia perdurans		2.12		1.55
Ostryopsis nobilis		2.01		
Trigonotis gracilipes		1.46		0.75
Tibetia yunnanensis		1.25		
Saussurea wardii		1.24	0.12	0.70
Saussurea leontodontoides		1.12		
Acanthocalyx nepalensis subsp. *delavayi*		0.88		
Tibetia tongolensis		0.82		0.35
Ophiopogon bodinieri		0.76		
Pedicularis elwesii		0.62	1.74	0.34

Rhodiola wallichiana	0.60		0.44
Juniperus saltuaria	0.59		
Thalictrum finetii	0.55		
Thalictrum atriplex	0.54		
Larix potaninii var. *macrocarpa*	0.53		0.18
Sedum multicaule	0.52		0.24
Acanthocalyx nepalensis	0.50		
Indigofera tinctoria	0.48		
Lilium lophophorum	0.47	0.04	
Campylotropis polyantha	0.46		
Cotoneaster multiflorus	0.44		
Ligusticopsis scapiformis	0.41	0.02	
Primula secundiflora	0.40		0.78
Trollius ranunculoides	0.35	0.14	0.12
Silene yunnanensis	0.31		
Saxifraga wardii	0.31		1.08
Rhodiola chrysanthemifolia	0.31		0.40
Saussurea montana	0.28		
Asplenium exiguum	0.24	0.73	0.09
Oxyria sinensis	0.24		0.13
Plantago asiatica subsp. *erosa*	0.23		0.45
Leontopodium leontopodioides	0.20	0.31	
Deutzia hookeriana	0.20		
Corydalis pachypoda	0.19	0.04	0.57
Ligularia cymbulifera	0.19		0.59
Meconopsis racemosa	0.15		
Tibetia himalaica	0.14		
Dolomiaea souliei	0.14		
Drynaria delavayi	0.12		
Sambucus adnata	0.10		
Cyclorhiza peucedanifolia	0.09		1.62
Rhodiola rosea	0.08		
Spiraea schneideriana	0.06		
Aster jeffreyanus	0.04	0.20	
Sophora davidii var. *chuansiensis*	0.04		
Pedicularis axillaris	0.03		
Codonopsis pilosula	0.02		
Cypripedium tibeticum	0.02		
Daphne longilobata	0.02		
Gentiana squarrosa	0.02		
Pedicularis siphonantha		3.75	
Rhododendron flavidum		2.87	
Saxifraga unguiculata		2.57	
Argentina leuconota		2.16	
Sibbaldia cuneata		1.91	
Dryopteris acutodentata		1.91	

Koenigia filicaulis	1.32	
Hylotelephium spectabile	1.06	
Gentianella pygmaea	0.92	
Gentianopsis paludosa	0.89	
Erysimum cheiranthoides	0.88	
Dolomiaea berardioidea	0.86	
Megacarpaea delavayi	0.84	
Saxifraga diversifolia	0.83	
Adonis davidii	0.82	
Sedum oreades	0.81	
Potentilla coriandrifolia var. *dumosa*	0.69	
Primula amethystina	0.60	
Ligularia tsangchanensis	0.58	
Rhododendron lapponicum	0.51	
Corydalis edulis	0.44	
Ribes tenue	0.43	
Micranthes pallida	0.33	0.45
Odontostemma glandulosum	0.29	
Ligusticopsis daucoides	0.25	
Draba nemorosa	0.19	
Rosa sweginzowii	0.16	0.48
Dasiphora fruticosa	0.15	
Aconitum carmichaelii	0.13	
Corydalis gracillima	0.11	
Koenigia pilosa	0.11	
Allium wallichii	0.11	
Juniperus indica	0.08	1.14
Astragalus acaulis	0.02	
Onychium siliculosum		3.86
Potentilla pamiroalaica		3.39
Circaeaster agrestis		3.30
Parnassia brevistyla		1.94
Chrysosplenium griffithii		1.31
Nardostachys jatamansi		1.03
Ixeris polycephala		0.80
Pedicularis lutescens		0.79
Rhododendron rupicola var. *chryseum*		0.65
Megacodon stylophorus		0.52
Microula sikkimensis		0.41
Clematis yunnanensis		0.30
Rhodiola fastigiata		0.20
Abies georgei var. *smithii*		0.20
Cardamine macrophylla		0.09
Silene chungtienensis		0.06
Anemone demissa		0.04

Table S6 Performance of models and variable contributions for each *Juniperus* species.

	AUC	variable contributions (%)							
		RM	FC	bio2	bio3	bio6	bio7	bio15	bio17
JSQ n=286	0.970± 0.01	0.1	LQ	0.3	7.2	28	58.1	2	4.4
JSA n=195	0.975± 0.02	3	H	0.4	13.8	30	41.1	8.7	6.1
JP n=208	0.982± 0.01	1.5	LQH	0.2	14.3	30.3	53.3	0.9	0.9
JI n=172	0.989± 0.01	0.1	LQ	0.2	40.1	26.8	20.6	0.3	12.1
JF n=479	0.950± 0.03	0.5	LQHP	8.8	0.5	26.3	52.7	10.6	1
JC n=117	0.991± 0.01	1.5	LQH	0.2	49	26	7.5	1.2	16.1
JPW n=50	0.992± 0.02	0.5	H	0.1	32.8	28.2	38.3	0.2	0.4

JSQ, *Juniperus squamata*, JSA, *Juniperus saltuaria*, JP, *Juniperus pingii*, JI, *Juniperus indica,* JF, *Juniperus formosana*, JC, *Juniperus coxii*, JPW, *Juniperus pingii* var. *wilsonii*. n= coordinate records.

Table S7. Predicted potential distribution areas of the seven *Juniperus* species for the present and two scenarios under future (2100) climate, and the overlap areas between the present and future.

	Suitability	Present ($\times10^4 km^2$)	BCC-CSM2-MR 126	Difference respect to present ($\times10^4$ km^2, %)	Overlap with present ($\times10^4 km^2$, %)	BCC-CSM2-MR 585	Difference respect to present ($\times10^4 km^2$, %)	Overlap with present ($\times10^4 km^2$, %)
	Total Predicted area	239.6	172.3	-67.3(-28.1)	166.6(69.5)	139.26	-100.4(-41.9)	119.5(49.9)
JSQ	Threshold - 0.4	134.3	89.1	-45.2(-33.7)		62.08	-72.2(-53.8)	
	0.4 - 0.6	64.6	50.3	-14.4(-22.2)		35.35	-29.3(-45.3)	
	0.6 - 1	40.7	33.0	-7.7(-19)		41.83	1.1(2.8)	
	Total Predicted area	184.8	154.8	-30(-16.2)	146.2(79.1)	159.52	-25.3(-13.7)	116.2(62.9)
JSA	Threshold - 0.4	108.0	86.2	-21.7(-20.1)		79.51	-28.4(-26.3)	
	0.4 - 0.6	43.7	39.9	-3.7(-8.6)		44.64	1(2.2)	
	0.6 - 1	33.2	28.7	-4.5(-13.7)		35.37	2.2(6.6)	
	Total Predicted area	127.7	96.6	-31.1(-24.3)	94.2(73.8)	83.84	-43.9(-34.4)	78.5(61.5)
JP	Threshold - 0.4	57.6	47.0	-10.6(-18.4)		33.98	-23.7(-41)	
	0.4 - 0.6	39.3	30.2	-9.1(-23.1)		25.71	-13.6(-34.5)	
	0.6 - 1	30.8	19.4	-11.4(-37.1)		24.15	-6.7(-21.6)	
	Total Predicted area	86.5	82.2	-4.3(-5)	77.8(89.9)	70.91	-15.6(-18.1)	61.8(71.4)
JI	Threshold - 0.4	53.7	51.0	-2.7(-5)		42.65	-11.1(-20.6)	
	0.4 - 0.6	24.7	23.0	-1.7(-7)		19.27	-5.4(-22)	
	0.6 - 1	8.1	8.2	0.1(1.2)		8.99	0.9(10.6)	
	Total Predicted area	431.8	393.3	-38.5(-8.9)	379.4(87.9)	316.92	-114.9(-26.6)	292(67.6)
JF	Threshold - 0.4	173.0	172.6	-0.5(-0.3)		202.55	29.5(17.1)	
	0.4 - 0.6	174.3	167.5	-6.8(-3.9)		88.96	-85.3(-49)	
	0.6 - 1	84.5	53.2	-31.2(-37)		25.41	-59(-69.9)	
	Total Predicted area	65.2	50.5	-14.7(-22.6)	49.4(75.8)	41.82	-23.3(-35.8)	35.7(54.8)
JC	Threshold - 0.4	37.4	34.1	-3.3(-8.7)		26.9	-10.5(-28)	
	0.4 - 0.6	22.1	13.9	-8.3(-37.3)		11.41	-10.7(-48.4)	
	0.6 - 1	5.7	2.5	-3.2(-56.3)		3.51	-2.2(-38.1)	
	Total Predicted area	105.2	90.5	-14.7(-14)	84.9(80.7)	100.48	-4.7(-4.5)	74.5(70.8)
JPW	Threshold - 0.4	51.8	40.9	-10.9(-21.1)		51.59	-0.2(-0.5)	
	0.4 - 0.6	29.4	29.5	0.1(0.4)		28.98	-0.4(-1.3)	
	0.6 - 1	24.0	20.1	-3.9(-16.1)		19.91	-4(-16.9)	

JSQ, *Juniperus squamata*, JSA, *Juniperus saltuaria*, JP, *Juniperus pingii*, JI, *Juniperus indica,* JF, *Juniperus formosana*, JC, *Juniperus coxii*, JPW, *Juniperus pingii* var. *wilsonii*. Threshold = 0.2

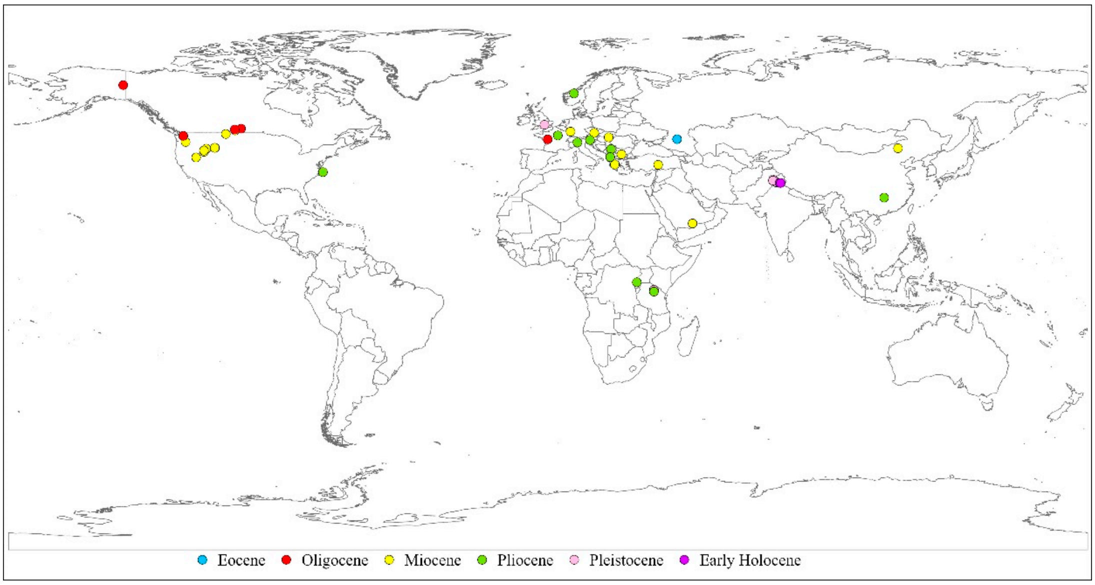

Figure S1 Fossils of *Juniperus* from the Paleogene, Neogene and Quaternary. The major sources documenting these fossil records are published journal articles, monographs and the paleobiological database (https://paleobiodb.org/)

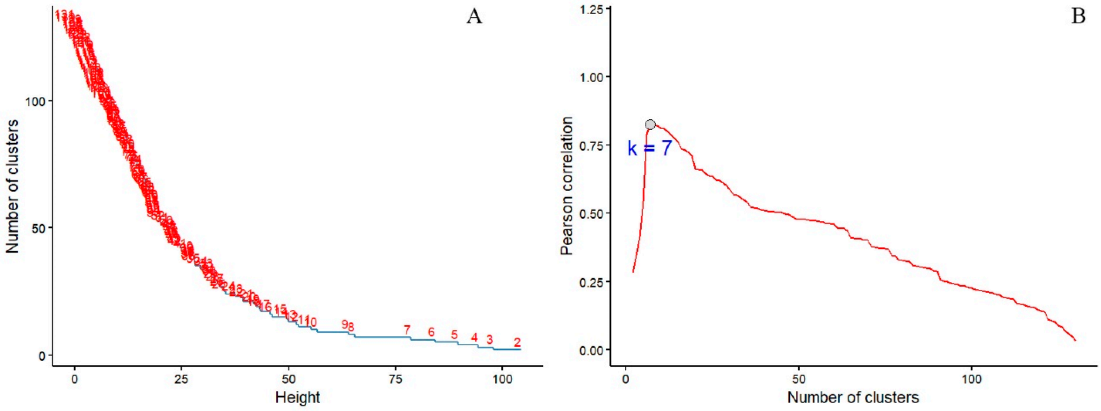

Figure S2 The best number of clusters for *Juniperus* community types. A: the fusion-level diagram of the dendrogram; B: the plot of Mantel statistic.

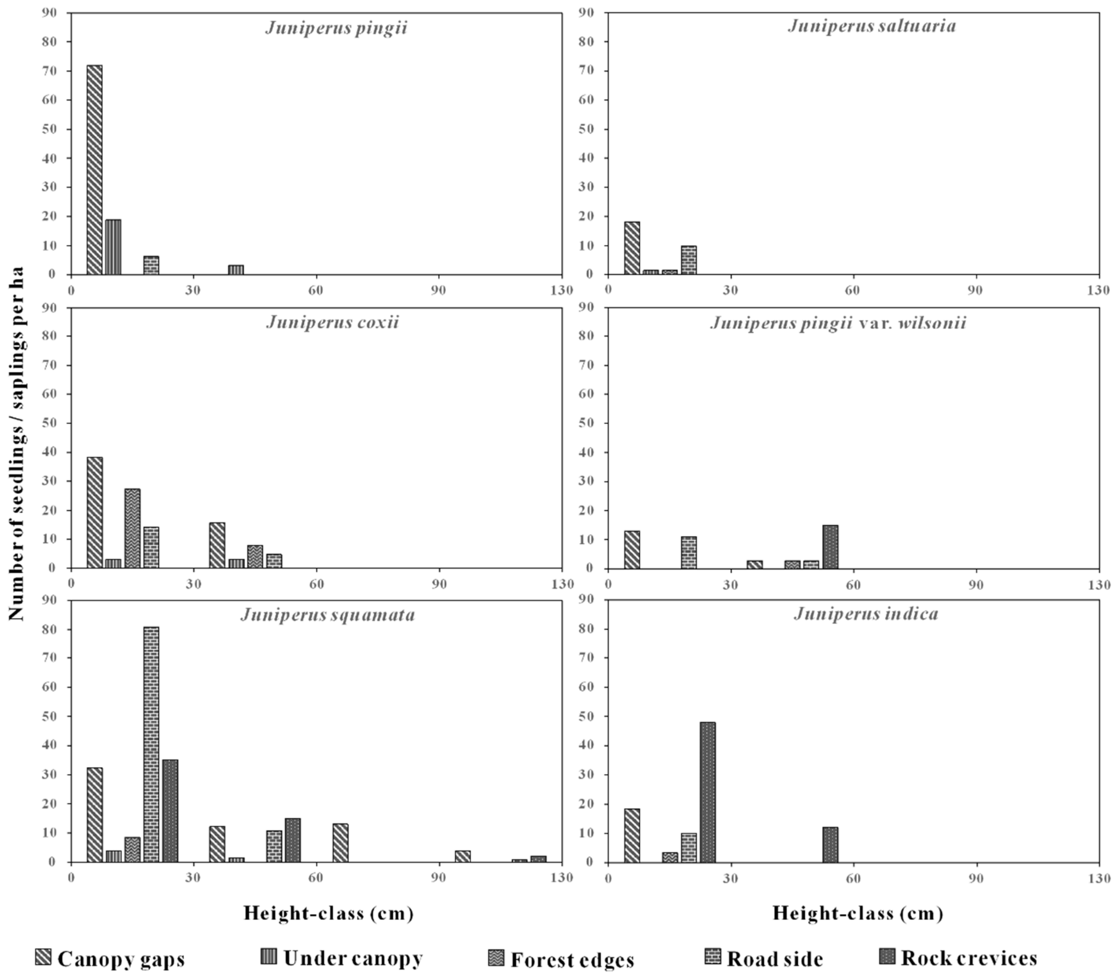

Figure S3. Variation in the density of juveniles and saplings of *Juniperus* species across different height classes in various micro-habitats.

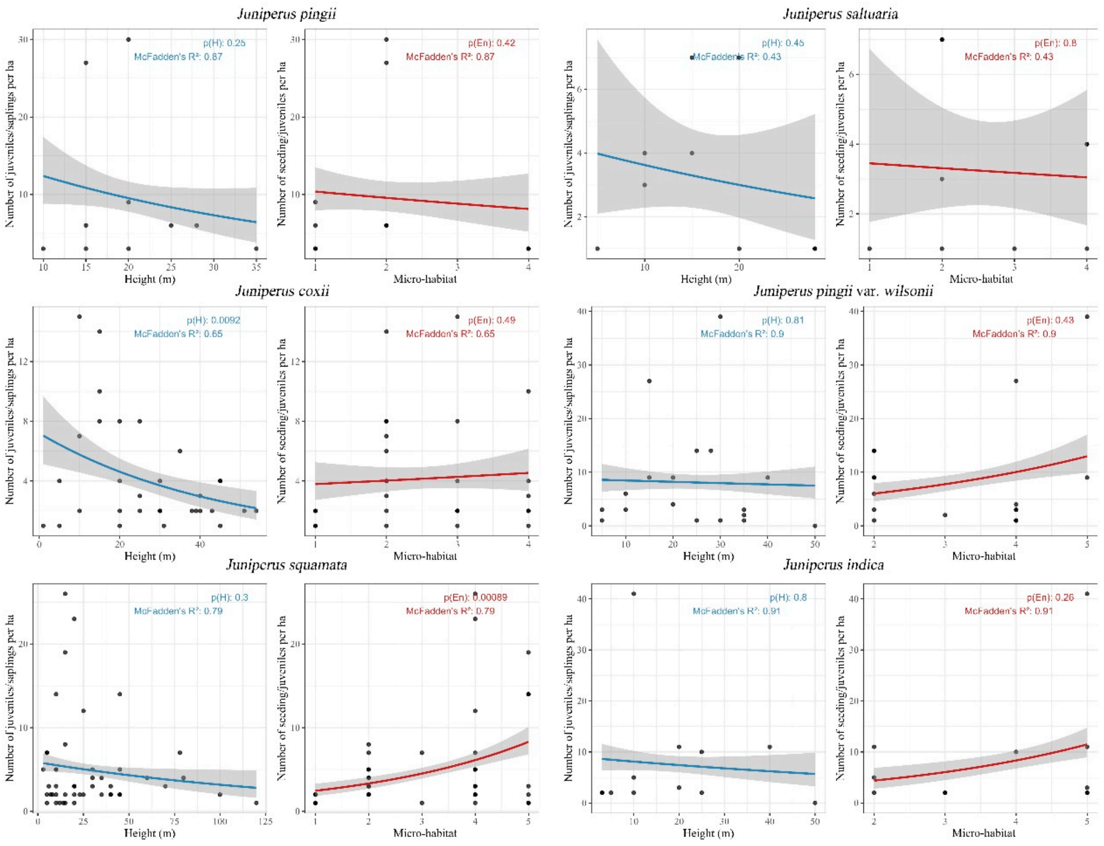

Figure S4 The GLM analysis of juveniles/saplings of *Juniperus* species. Left (blue): between different height and the density of juveniles and saplings; Right (red): between various micro-habitats and the density of juveniles/saplings. Micro-habitats: 1, Under canopy; 2, Canopy gaps; 3, Forest edges; 4, Road side; 5, Rock crevices.

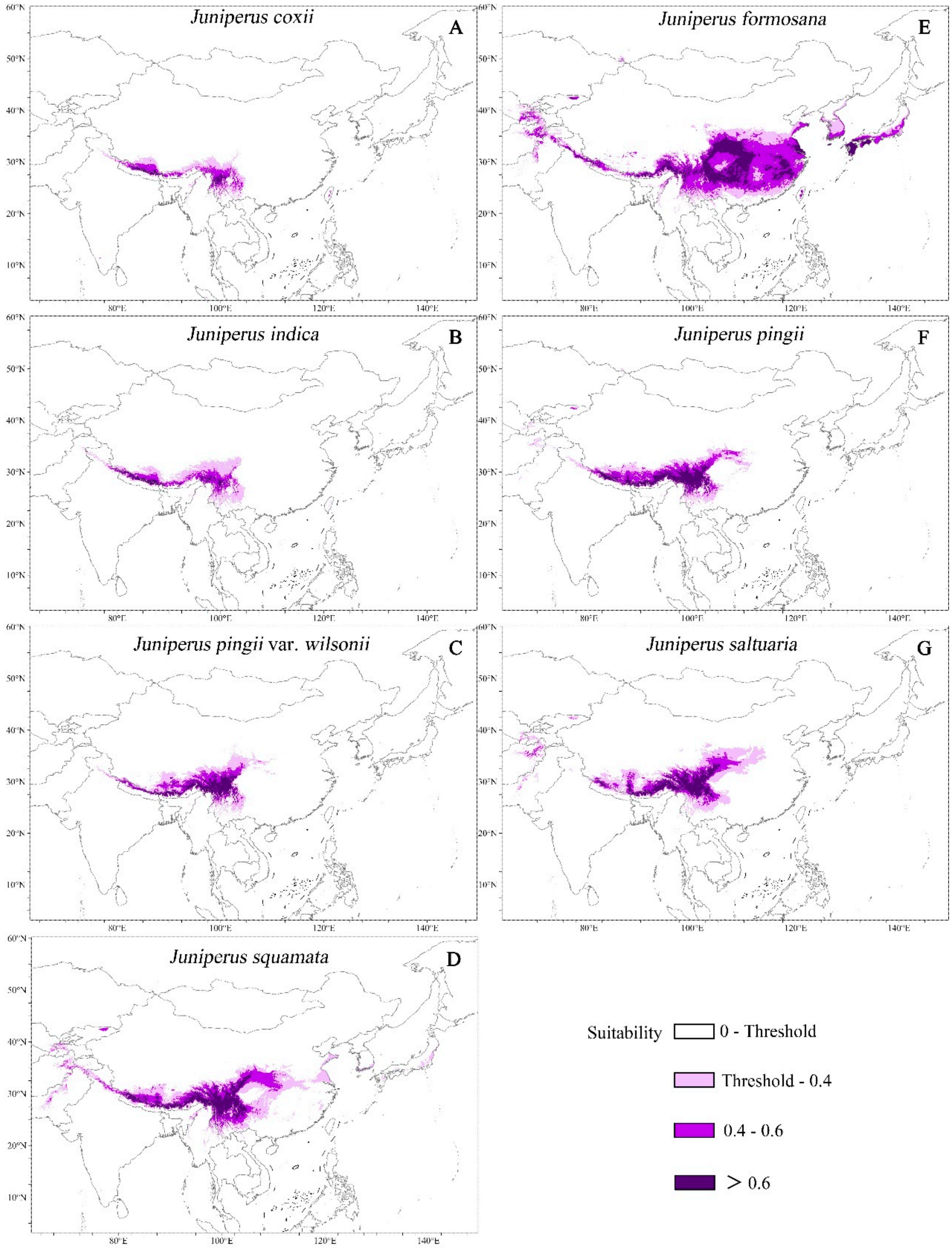

Figure S5. The potential habitats under the present climate scenarios. (A)-(G): Potential habitats of the seven species in the present.

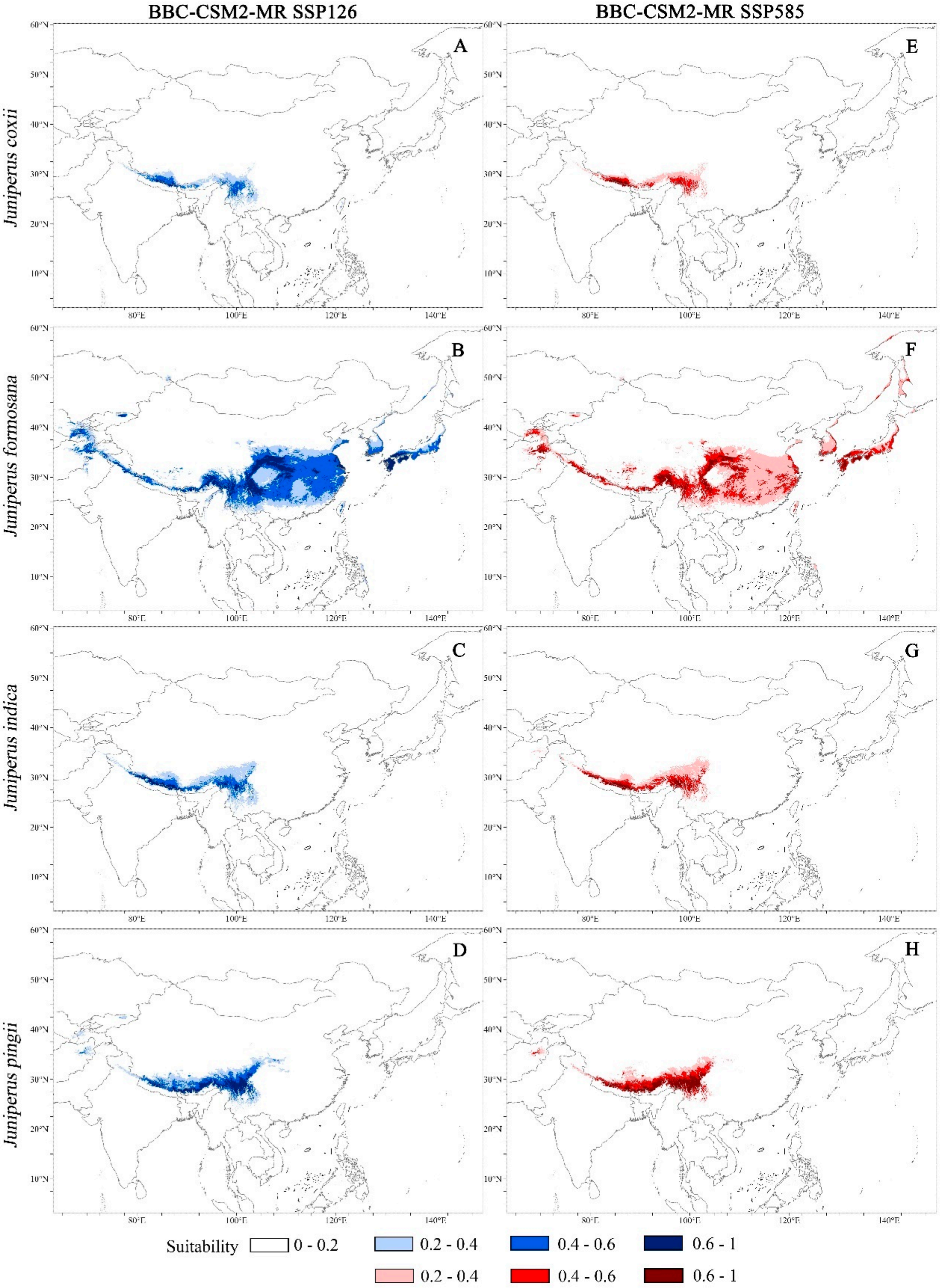
BBC-CSM2-MR SSP126
BBC-CSM2-MR SSP585
Juniperus coxii
Juniperus formosana
Juniperus indica
Juniperus pingii
A
B
C
D
E
F
G
H
60°N
50°N
40°N
30°N
20°N
10°N
80°E
100°E
120°E
140°E
Suitability
0 - 0.2
0.2 - 0.4
0.4 - 0.6
0.6 - 1
0.2 - 0.4
0.4 - 0.6
0.6 - 1

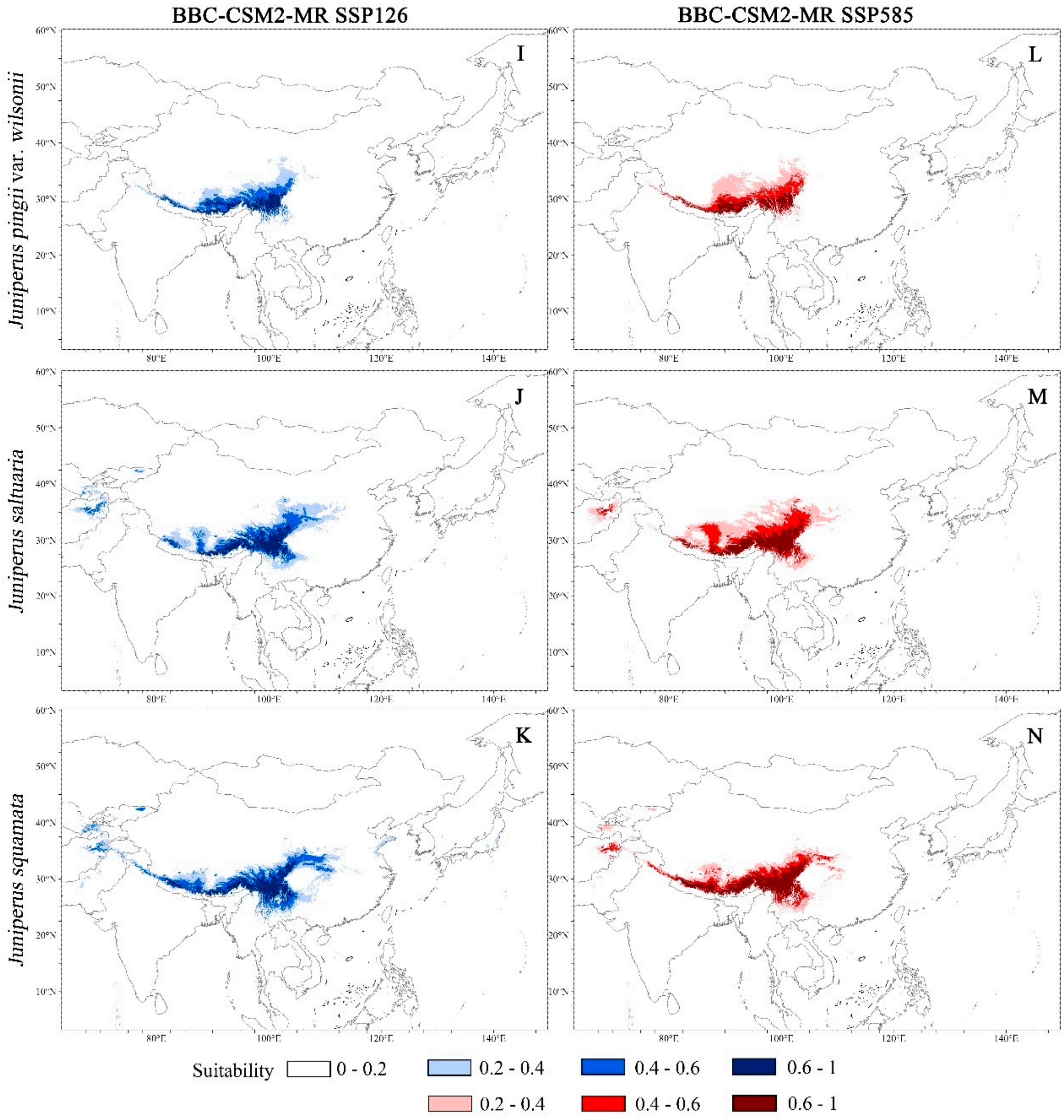

Figure S6. The potential habitats under the two climatic scenarios in the future (2100). (A)-(D) and (I)-(K): Potential habitats under the scenarios 2100-BCC-CSM2-MR SSP126; (E)-(H) and (L)-(N): Potential habitats under the scenarios 2100-BCC-CSM2-MR SSP585.
